# Jianpi Yangwei decoction promotes apoptosis and suppresses proliferation of 5-fluorouracil resistant gastric cancer cells in vitro and in vivo

**DOI:** 10.1186/s12906-020-03135-8

**Published:** 2020-11-10

**Authors:** Huijuan Tang, Wenjie Huang, Qiang Yang, Ying Lin, Yihui Chen, Peng Shu

**Affiliations:** 1 Oncology Department, Affiliated Hospital of Nanjing University of Chinese Medicine, 155 Hanzhong Road, Nanjing, 210029 Jiangsu province China; 2 Department of Clinical and Molecular Sciences, Università Politecnica delle Marche, 60126 Ancona, Italy; 3 Oncology Department, Liyang Traditional Chinese Medicine Hospital, Liyang, 213300 China; 4 Emergency Department, Suzhou Municipal Hospital of Integrative Medicine, Suzhou, 215000 China; 5 Gastroenterology Department, Jiangsu Jiangyin People’s Hospital, Jiangyin, 214400 China

**Keywords:** Jianpi Yangwei decoction, Gastric cancer, Drug-resistance, PI3K/AKT pathway

## Abstract

**Background:**

The exploration of new therapeutic agents targeting 5-Fu resistance may open a new opportunity to gastric cancer treatment. The objective is to establish a 5-Fu resistant gastric cancer cell line and observe the effect of Jianpi Yangwei decoction (JPYW) on its apoptosis and drug-resistance related proteins.

**Methods:**

MTT assay was used to measure the effect of JPYW on the BGC823 cells proliferation, and the apoptosis was observed by flow cytometry and Hoechst fluorescence staining. The BGC823 xenograft tumor nude mice models were established, the apoptosis was detected by Tunel method. BGC-823/5-Fu was established by repeated low-dose 5-Fu shocks, the drug resistance index and proliferation were detected by the MTT assay; MDR1 mRNA was detected by real-time RT-PCR; Western blot was used to detect the ratio of p-AKT to AKT; The BGC823/5-Fu xenograft tumor nude mice models were established and apoptosis was measured. The expressions of MRP1, MDR1, ABCG2, AKT, p-AKT, caspase-3 and bcl-2 were detected by immunohistochemistry and the AKT mRNA expression was detected by real-time RT-PCR.

**Results:**

JPYW induced apoptosis in BGC823 cells; Drug-resistant cell line BGC-823/5-Fu was sucessfully established; JPYW induced apoptosis of BGC823/5-Fu cells, down-regulated the expression of MRP1, MDR1 and ABCG2 in vitro and in vivo, and further decreased MDR1 expression when combined with pathway inhibitor LY294002 (*P* < 0.05); JPYW down-regulated the ratio of p-AKT to AKT in vitro in a dose-dependent manner, the same as after the combination with LY294002 (P < 0.05).

**Conclusion:**

JPYW can induce apoptosis of BGC823 and BGC823/5-Fu cells, and down-regulate the expression of MDR1, MRP1, ABCG2 in vitro and in vivo. Its in vitro effect is related to the PI3K/AKT signaling pathway.

**Supplementary Information:**

The online version contains supplementary material available at 10.1186/s12906-020-03135-8.

## Background

Gastric cancer remains one of the most common malignant tumors worldwide, especially in China [1]. Although the overall morbidity of gastric cancer has been declined globally in recent years, the number of patients is still huge. Chemotherapy is the main treatment method of gastric cancer. 5-fluorouracil (5-Fu) and its derivatives are the cornerstones of standard chemotherapy regiments for gastric cancer. A growing body of evidence suggests the outstanding efficacy with a combination of 5-Fu and other anti-tumor drugs in gastric cancer [2]. A meta analysis included 64 RCTs demonstrated that first-line combination chemotherapy improves survival compared to single-agent 5-Fu. And the addition of docetaxel to platinum-fluoropyrimidine-based chemotherapy regimens appears to extend survival rate extremely [3]. However, the effective rate of chemotherapy is restricted and the application of new drugs and regimens has not led to fundamental improvements in its efficacy [4]. It is largely attributed to the existence of multi-drug resistance of gastric cancer cells, which caused less sensitivity to chemotherapy. A crowd of gastric cancer patients are suffered from acquired progressive 5-Fu resistance after the initial curative effects [5]. Since chemotherapies with 5-Fu have remarkable effectiveness and its drug resistance reduces the chemotherapy effective rate, the exploration of new therapeutic agents targeting 5-Fu resistance may bring an opportunity to break the bottleneck of current treatment status.

It is well known that multidrug resistance in gastric cancer treatment involves a large number of molecular targets as well as complex mechanisms [6]. The formation of multidrug resistance notably reduced the chemotherapy efficacy [7]. Classic drug resistant proteins, including multidrug resistance gene 1 (MDR1), multidrug resistance-associated protein 1 (MRP1) and ATP binding cassette subfamily G member 2 (ABCG2) recently attract attentions in gastric cancer researches [8]. MDR1, MRP1 and ABCG2 are part of the ATP-binding cassette (ABC) subfamily, which regulate the efflux of drugs out of cells and reduce treatment efficacy.

In terms of reversing drug-resistance, although many drugs have definite effects in vitro experiments, but they are not suitable for clinical use due to their large side effects. Recently, the use of natural products for treating tumors has gained increasing attention [9]. Among them, Traditional Chinese Medicine (TCM) has become a hot topic because of its low toxicity and excellent effectiveness [10]. TCM has been recognized significant advantages in alleviating the clinical symptoms and the side effects of chemotherapy, and improving the chemotherapy efficacy [11, 12]. Jianpi Yangwei decoction (JPYW) was created by professor Shenlin Liu through summarizing decades of experience on diagnosis and treatment of gastric cancer. The curative effect of this prescription has been proved by clinical observations and basic experiment studies by our previous researches The clinical observation on 489 II, III stage gastric cancer patients in the State Administration of Traditional Chinese Medicine multi-center clinical research program (NO.200807022) demonstrated that the risk of recurrence was decreased by 32.8% compared with chemotherapy alone (*P* = 0.0042) [13]. However, the underlying mechanism of JYPW has not been fully investigated. In this research, we established a drug-resistant cell line of human gastric cancer, and observed the effect of JPYW on its apoptosis and drug-resistant proteins in vitro and in vivo so as to provide the theoretical and experimental basis for JPYW in gastric cancer treatment. Since JPYW needs to be taken orally by patients in clinical practice, we verified the anti-tumor effects through animal experiments to make the results more reliable.

## Methods

### Materials

Human gastric cancer BGC823 cells were purchased from ATCC. Nude mice were purchased from Berke Biology co. LTD. China. JPYW was formed by *Astragalus propinquus* 15 g, *Codonopsis pilosula* 15 g, *Atractylodes macrocephala* 10 g, *Angelica sinensis* 10 g, *Paeonia lactiflora* 10 g, the pericarp of *Citrus reticulata* 6 g, ginger processed *Pinellia ternata* 10 g, *Sparganium stoloniferum* 10 g, *Curcuma zedoaria* 10 g, *Salvia chinensis* 30 g, *Hedyotis diffusa* 30 g, and *Liquiritia* 5 g*.* The abovementioned traditional Chinese medicinal materials were used to prepare a 400-ml decoction by the pharmaceutical department of Jiangsu Provincial Hospital of TCM. After filtraltion and concentration the decoction, we obtained JPYW stock with a concentration of 2.0 g/mL. Culture medium was used to dilute the stock to relevant concentration for experiments in vitro, while PBS was used to dilute the stock to relevant concentration for experiments in vivo. MRP1, MDR1, ABCG2, AKT, p-AKT, caspase-3 and bcl-2 antibodies were purchased from Cell Signaling Technology, Inc. USA. LY294002 was purchased from Sigma Aldrich Co. USA. All other reagents used were of analytical grade.

### MTT assay

2 × 10^3^ BGC823 or 4 × 10^3^ BGC823/5-Fu cells were seeded in 96-well plates. Cells in each group were cultured with the corresponding concentration of JPYW or 5-Fu before reacting with MTT reagent. DMSO was added to dissolve formazan, and then the OD value was measured.

### Hoechst fluorescence staining

Cells were cultured in six-well plates containing glass cover slips. JPYW was added when 70% of the cells adhered to the glass cover slips. After 48 h, the glass cover slips were then washed with PBS, fixed with 4% formaldehyde and washed again. Then, cells were incubated at the room temperature for 30 min with the Hoechst 33342 solution. The solution was removed and cells were observed under a fluorescence microscope.

### Flow cytometry

BGC823 cells were seeded in petri dishes, and were divided into groups. After cultured with JPYW for 48 h, Annexin-V/PI staining were added in sequence. Flow cytometry detection was used for analysis.

### BGC823/5-Fu establishment

BGC823 cells were cultured with medium contain 5-Fu for 1 h. Then cells were transferred to a new culture flask and the medium was changed next day. The cells were continuedly cultured with 5-Fu until there is no longer mass death, with the concentration of 5-Fu increased time by time until 10 μg/ml. The morphology and drug resistance index (RI) of the cells were observed.

### Treatment of JPYW on BGC823 and BGC823/5-Fu cells in vivo

In the animal laboratory, 25 nude mice (male; 7 weeks old; 20-22 g weight; fed inIsolator with sterilized wood shavings bedding, mouse feed and water; regular light for 10 h per day) got subcutaneous injection with 2 × 10^7^ BGC823 or BGC-823/5-Fu cells respectively and the tumor volumes were measured weekly. After two weeks, according to clinical methods and metabolic rate of nude mice, 25 mice were randomly divided into Control group, JPYW-low group (14 g·kg^− 1^·d^− 1^), JPYW-high group (28 g·kg^− 1^·d^− 1^), 5-Fu group, JPYW+ 5-Fu group (JPYW 28 g·kg^-1^·d^-1^), 5 mice in each group. 5-Fu was intraperitoneally injected as 25 mg·kg^− 1^ once two days. JPYW was intragastric administrated as 10 ml·kg^− 1^ twice a day. The mice were sacrificed by cervical dislocation method and the tumors were taken after being treated for 14 days, then apoptosis was observed by Tunel method, the expressions of MRP1, MDR1, ABCG2, AKT, p-AKT, caspase-3 and bcl-2 were observed by immunohistochemistry. The AKT mRNA were detected by real-time RT-PCR.

### Western blot

After cells were cultured with JPYW, cell proteins were extracted and the protein concentrations were determined by Braford method. The expression of AKT and p-AKT in each group was detected by western blot method, and the grayscale values were observed.

### Real-time RT-PCR

Cells were collected for real-time RT-PCR detection. Primer sequences were as follows:

- β-actin-F(CAGTCGGTTGGAGCGAGCAT);
- β-actin-R(GGACTTCCTGTAACAACGCATCT);
- MDR1-F(CTGTTGGCGTATTTGGGATGT);
- MDR1-R(CAGCATCAAGAGGGGAAGTAATG);
- AKT-F(ATGAACGACGTAGCCATTGTG);
- AKT-R(TTGTAGCCAATAAAGGTGCCAT) .

### Statistic analysis

SPSS 19.0 software was used for statistical analysis, and all data were represented by M ± S (mean ± standard deviation). *P* < 0.05 was considered as statistically significant. T test was used for comparison between two groups. One-way ANOVA test was used for comparison between multiple groups.

## Results

### JPYW induced BGC823 apoptosis

**Fig. 1:**
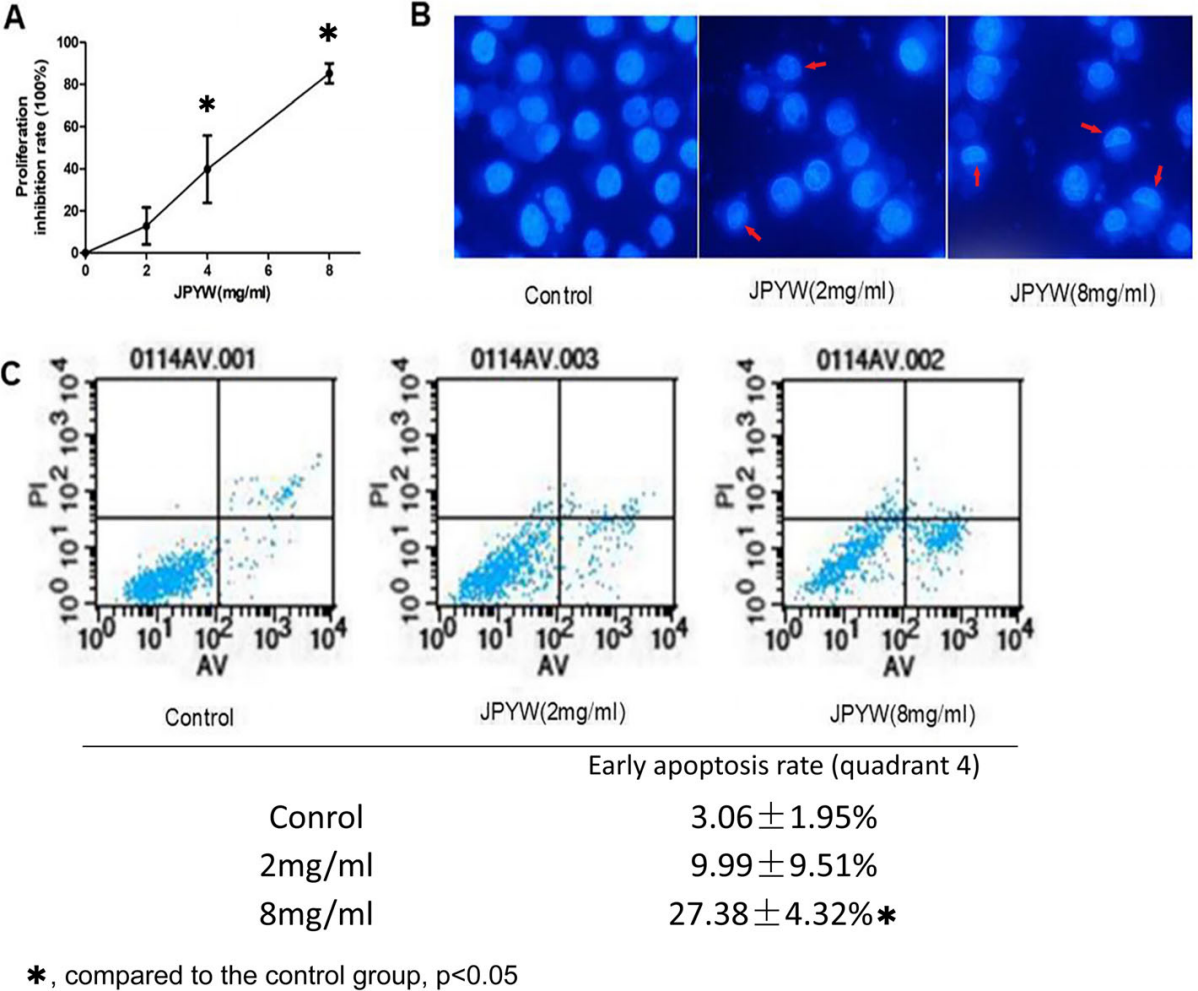
JPYW induced BGC823 cells apoptosis. **a**. MTT assay showed a dose-dependent cell proliferation inhibition of JPYW on BGC823 cells; **b**. Staining with Hoechst 33342, the control BGC823 cells showed normal nuclear morphology, and few apoptotic cells with nuclear condensation. The cells treated with JPYW showed increased number of apoptotic cells with obvious cytoplasmic wrinkling and nuclear edge aggregation, and formed obvious lunate corpuscles. (Magnification 400×); **c**. Flow cytometry showed that, following treatment with JPYW at 8 mg/ml, the percentage of apoptotic cells was found significantly increased as compared with the control group

To investigate the effect of JPYW on cell proliferation, the MTT assay was performed on BGC823 cells. Our preliminary experiment results showed that the 50% inhibitory concentration (IC_50_) of JPYW on BGC823 was 4.23 mg/ml, so we chose 2, 4, 8 mg/ml as the appropriate experimental concentrations. As shown in Fig. 1a, a dose-dependent cell proliferation inhibition on BGC823 cells was observed after treated with JPYW at 2, 4, 8 mg/ml for 48 h. The characteristic morphological changes were assessed by fluorescence staining with Hoechst 33,342. As shown in Fig. 1b, the control group showed normal nuclear morphology (blue) after stained with Hoechst 33342, and few apoptotic cells with nuclear condensation were observed. By contrast, the cells treated with JPYW (both 2 mg/ml and 8 mg/ml) showed increased number of apoptotic cells which formed obvious lunate corpuscles with cytoplasmic wrinkling and nuclear edge aggregation. The Annexin-V/PI double staining assay was conducted to evaluate cell apoptosis. Consistent with the results of Hoechst 33342 staining, as shown in Fig. 1c, after treated with JPYW at 8 mg/ml, the proportion of apoptotic cells increased significantly compared with the control group. All these results suggested that JPYW conspicuously induced apoptosis in BGC823 cells.

### Antitumor effects of JPYW in vivo

**Fig. 2:**
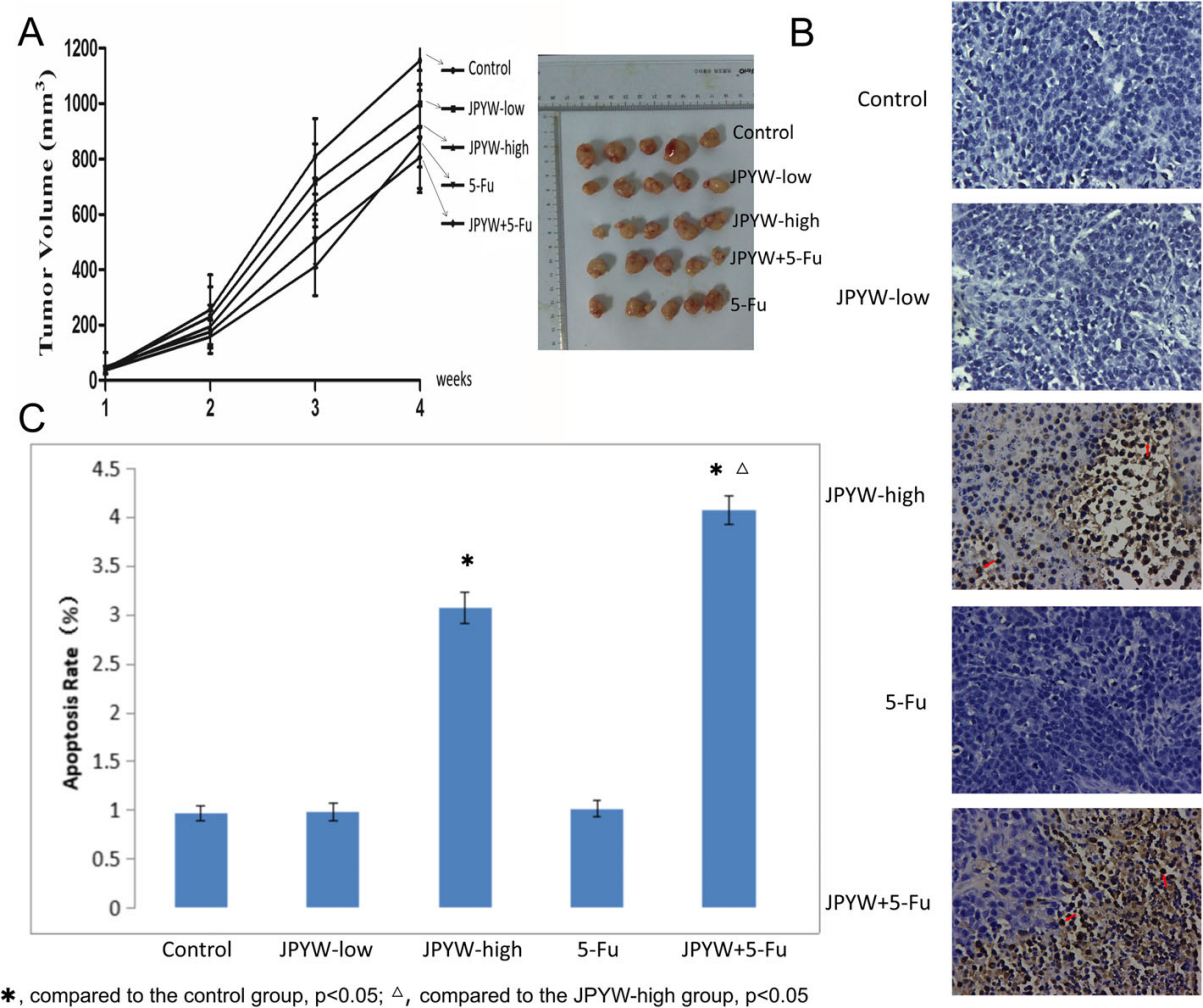
Antitumor effects of JPYW in vivo. **a**. Comparing the tumor volumes of mice, the JPYW-high group, 5-Fu group and JPYW+ 5-Fu group showed significant inhibitory effect on the growth of BGC823 tumor (*P* < 0.05), while the JPYW-low group showed not; **b**. Tunel apoptosis detection showed that JPYW-high group and JPYW+ 5-Fu group had dramatically increased numbers of TUNEL-positive cells. (Magnification 200×); **c**. The Tunel results showed that the apoptosis rates of the JPYW-high group and JPYW+ 5-Fu group were significantly increased compared with the control group, and the JPYW+ 5-Fu group showed higher apoptosis rate compared with JPYW-high group (*P* < 0.05)

The in vivo antitumor effects of JPYW were analyzed in BGC823 xenograft tumor model based on nude mice, the included numbers in each group is 5/5. JPYW significantly inhibited the growth of xenograft tumors compared with the control group. As shown in Fig. 2a, the JPYW-high group and JPYW+ 5-Fu group demonstrated significant inhibitory effect on the BGC823 xenograft tumor volumes(*P* < 0.05), while the JPYW-low group did not show this phenomenon. The tumor growth inhibition of the JPYW-high group and the 5-Fu group was similar and the JPYW+ 5-Fu group presented the most notably inhibitory effect. As shown in Fig. 2b and c, Tunel apoptosis detection showed that the apoptosis rates of the JPYW-high group and JPYW+ 5-Fu group were significantly increased, and the JPYW+ 5-Fu group showed higher apoptosis rate compared with JPYW-high group (P < 0.05), while there was no significant difference between the JPYW-low group and control group.

### BGC823/5-Fu establishment

**Fig. 3:**
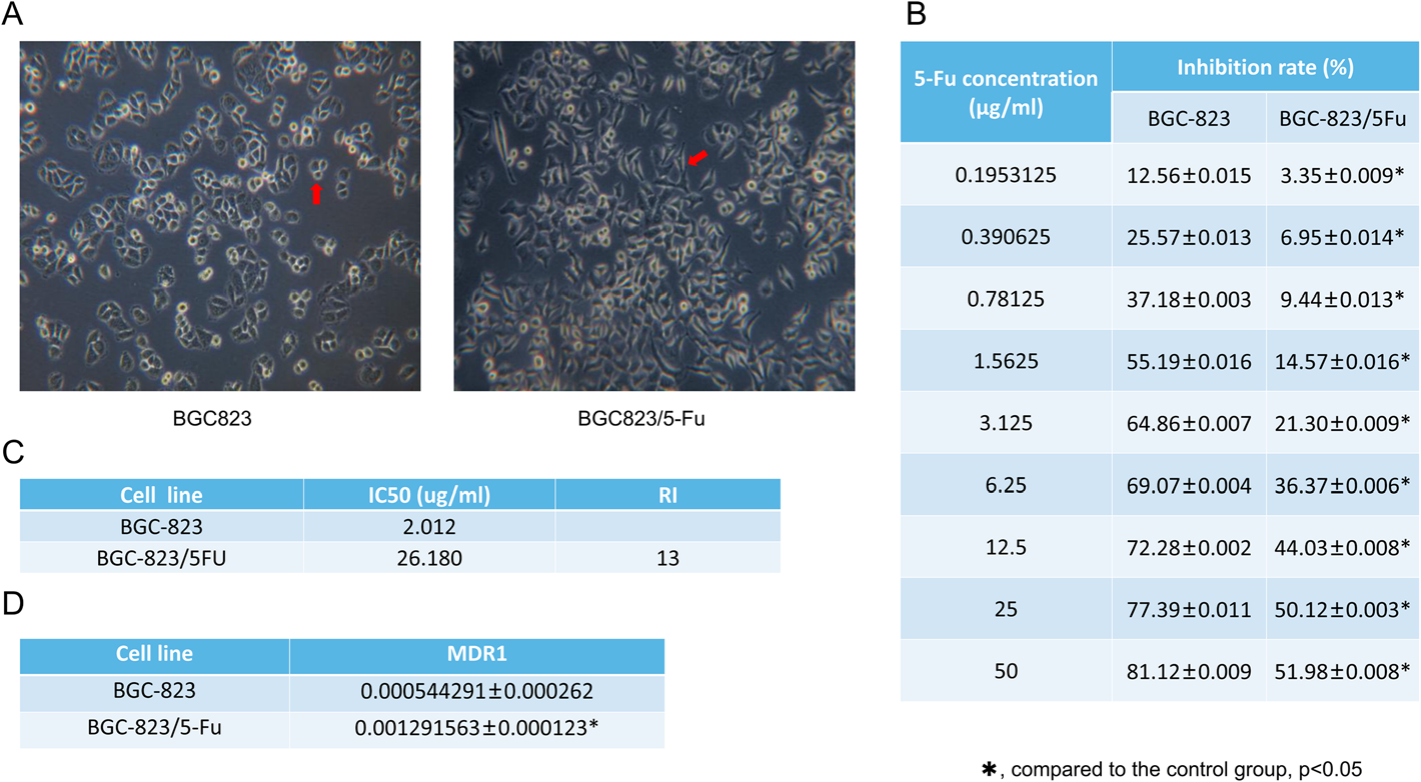
BGC823/5-Fu establishment. **a**. BGC823 cells were oval with clear boundary, uniform size, uniform cytoplasm and few irregular cells. BGC823/5-Fu cells were fusiform and more irregular in shape. (Magnification 400×); **b**. MTT assay showed that, with the increase of 5-Fu concentration, the inhibition rate of sensitive cells and drug-resistant cells both increased, but the inhibition rate of drug-resistant cells was lower than sensitive cells at the same concentration; **c**. The IC_50_ of 5-Fu on BGC823/5-Fu cells was 26.180 μg/ml, while on BGC823 was 2.012 μg/ml, thus, the drug RI of BGC823/5-Fu was 13 compared with BGC823; **d**. The real-time RT-PCR detection showed that the mRNA expression of MDR1 in BGC823/5-Fu cells (0.001291563 ± 0.000262) was significantly higher than BGC823 cells (0.000544291 ± 0.000123) (*P* < 0.01)

As shown in Fig. 3a, BGC823 cells appeared having oval shape with clear boundary, uniform size, uniform cytoplasm and few cells with irregular shape. BGC823/5-Fu cells were fusiform in shape and having more irregular shape cells. With the increase of 5-Fu concentration, the inhibition rate of sensitive cells BGC823 and drug-resistant cells BGC823/5-Fu both increased, but the inhibition rate of drug-resistant cells was lower than sensitive cells at the same concentration. (Fig. 3b). And the drug RI of BGC823/5-Fu was 13 (Fig. 3c). The real-time RT-PCR detection showed that the mRNA expression of MDR1 in BGC823/5-Fu cells (0.001291563 ± 0.000262) was significantly higher than BGC823 cells (0.000544291 ± 0.000123) (*P* < 0.01), as shown in Fig. 3d.

### Effect of JPYW on BGC823/5-Fu in vitro

**Fig. 4:**
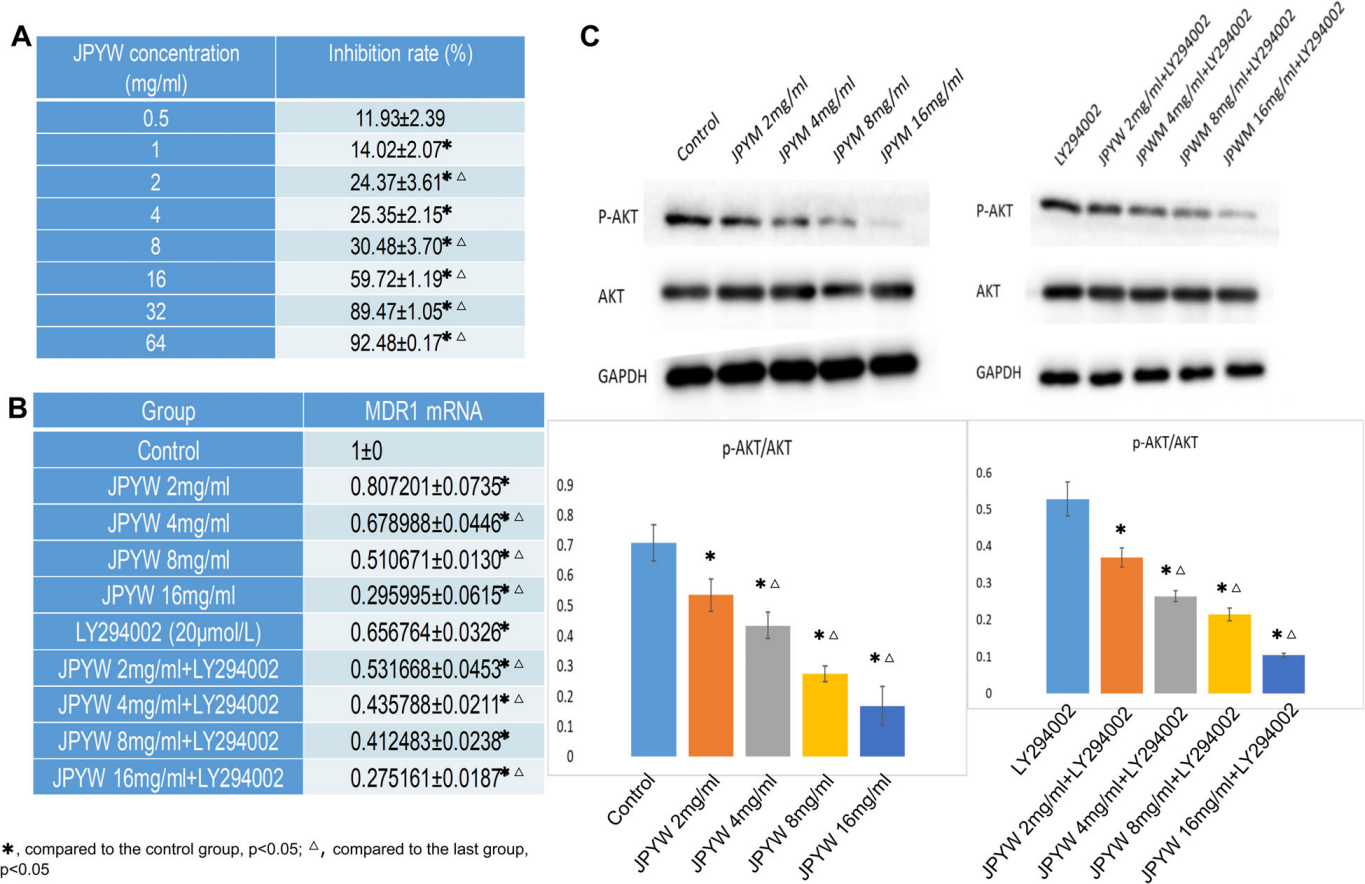
Effect of JPYW on BGC823/5-Fu in vitro. **a**. MTT assay showed that JPYW had a proliferation inhibition effect on BGC823/5-Fu, and the inhibition effect increased with the increase of concentration; **b**. The expression of MDR1 mRNA gradually decreased after JPYW treated BGC823/5-Fu cells, and the inhibitory effect was enhanced when combined with LY294002; **c**. The ratio of p-AKT expression and AKT expression dose-dependent decreased after the treatment of JPYW, also when combined with pathway inhibitor LY294002 (P < 0.05)

JPYW had an inhibitory effect on BGC823/5-Fu cell proliferation, and the inhibitory effect increased with the increase of concentration. The IC_50_ value of JPYW on BGC823/5-Fu cells was 7.32 ± 1.75 mg/ml, as shown in Fig. 4a. The MDR1 expression of BGC823/5-Fu cells gradually decreased with JPYW concentration increased. And the inhibitory effect was enhanced when combined with PI3K/AKT pathway inhibitor (LY294002), as shown in Fig. 4b. The expression level of p-AKT and AKT were detected by western blot. As shown in Fig. 4c, the ratio of p-AKT expression to AKT expression had a dose-dependent decrease relationship with the treatment of JPYW, and the same was true in the case of combined use with pathway inhibitor LY294002 (*P* < 0.05).

### Effect of JPYW on BGC823/5-Fu in vivo

**Fig. 5:**
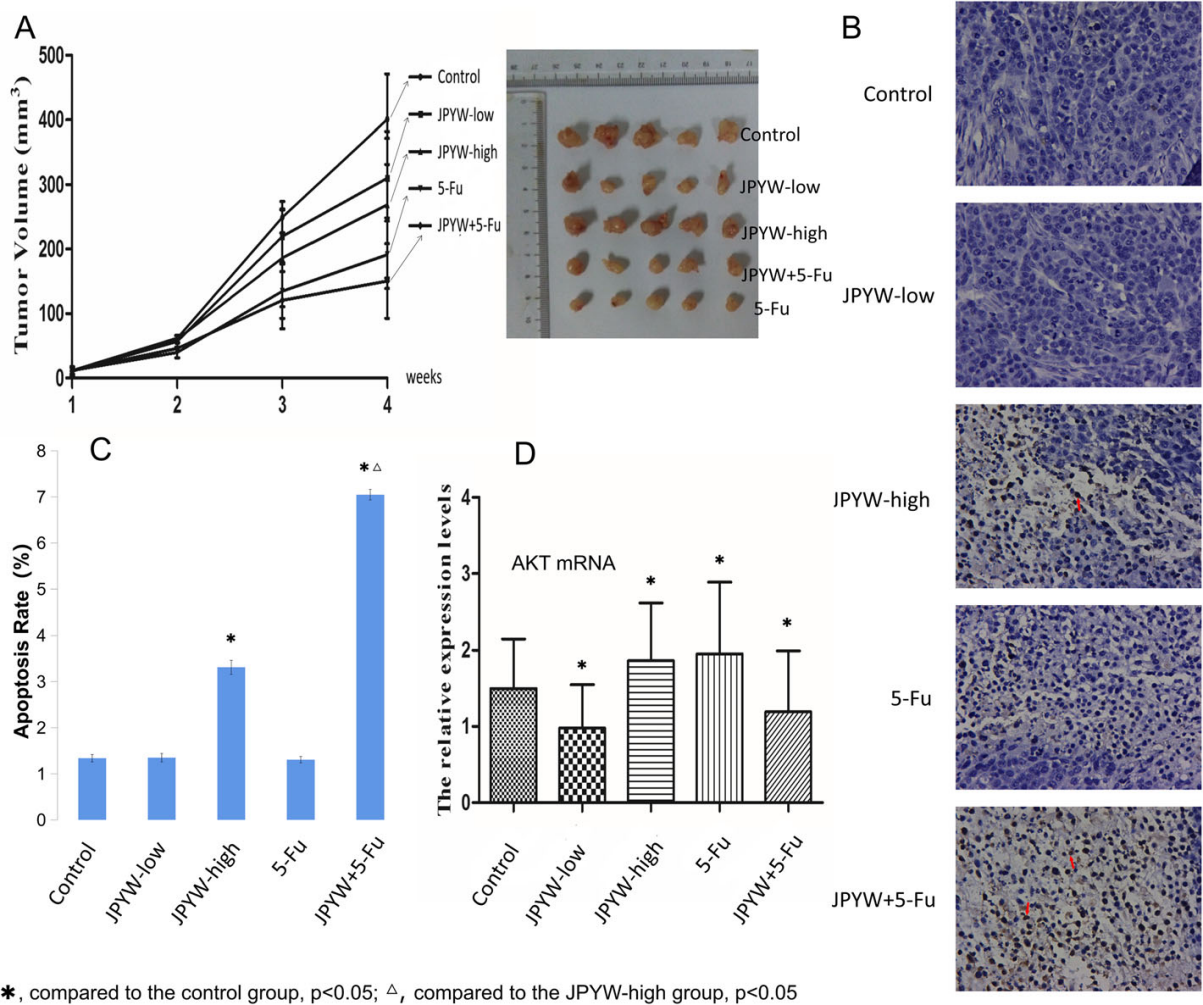
JPYW induced BGC823/5-Fu apoptosis in vivo. **a**. Comparing the tumor volumes of mice, the growth inhibition of the JPYW-high group was obvious and the JPYW+ 5-Fu group showed the most obvious inhibitive effect of tumor growth, while there was no significant difference between the JPYW-low group and the control group; **b**. Tunel apoptosis detection showed that the JPYW-high group and JPYW+ 5-Fu group had dramatically increased numbers of TUNEL-positive cells. (Magnification 200×); **c**. The Tunel results showed that the apoptosis rates of the JPYW-high group and JPYW+ 5-Fu group were significantly increased compared with the control group, and the JPYW+ 5-Fu group showed higher apoptosis rate compared with JPYW-high group (P < 0.05); **d**. In the JPYW-low group and JPYW+ 5-Fu group, the expression of AKT mRNA was significantly decreased, while in the JPYW-high group and 5-Fu was significantly increased

As shown in Fig. 5a, it was obvious that the JPYW-high group had an in vivo growth inhibition effect on BGC823/5-Fu xenograft tumors and the JPYW+ 5-Fu group showed the most conspicuously inhibitory effect, while there was no significant difference between the JPYW-low group and the control group. As shown in Fig. 5b and c, compared with control group, the apoptosis rate of the JPYW-high group was remarkably increased, and was further increased in the JPYW+ 5-Fu group (*P* < 0.05), while that of the JPYW-low group had no significant difference. As shown in Fig. 5d, in the JPYW-low group and JPYW+ 5-Fu group, the expression of AKT mRNA was significantly decreased, while in the JPYW-high group and 5-Fu group it was significantly increased.

**Fig. 6:**
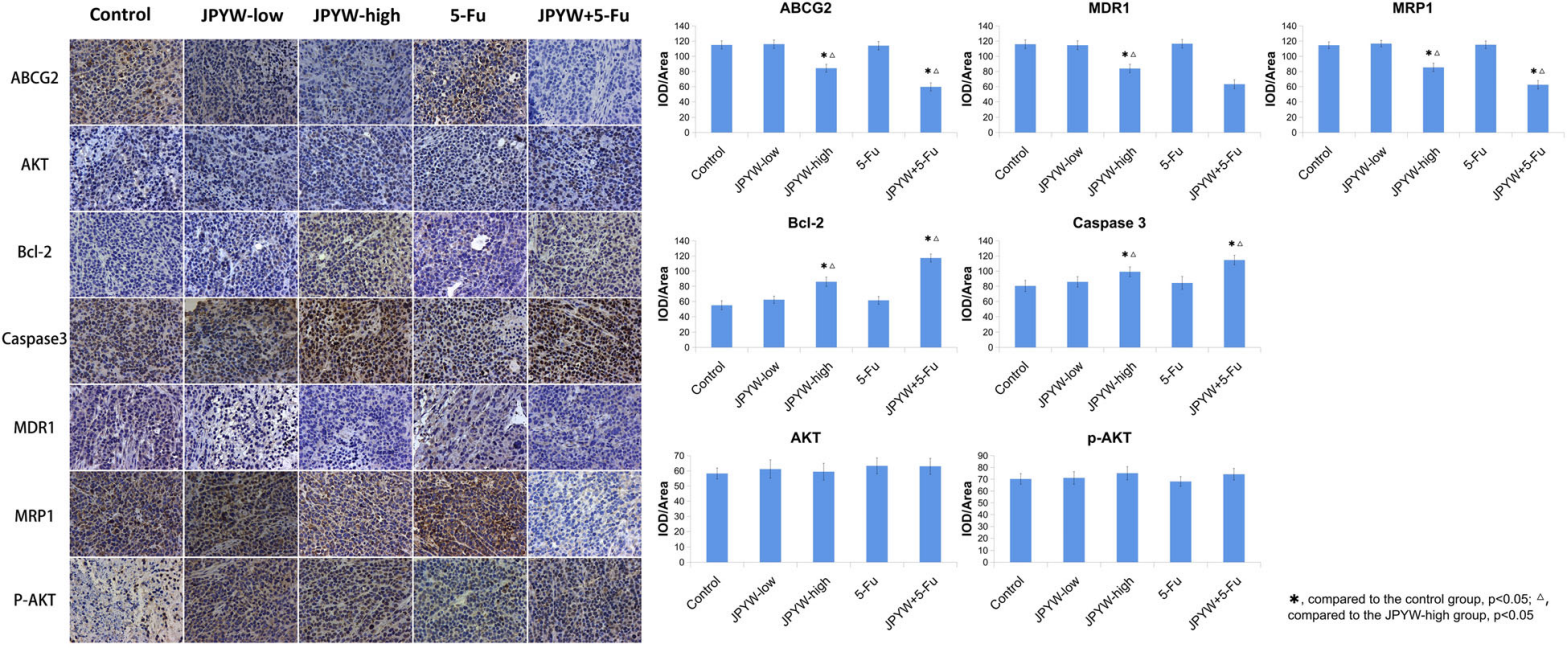
Immunohistochemical staining of BGC823/5-Fu tumors (Magnification 200×). Immunohistochemical staining of BGC823/5-Fu tumors was performed after the mice were treated for 14 days. As shown in the bar chart: Compared with the control group, the levels of ABCG2, MDR1 and MRP1 in JPYW-high group were significantly decreased, while the levels of Bcl-2, Caspase 3 were significantly increased (P < 0.05). Compared with the JPYW-high group, JPYW+ 5-Fu treatment significantly inhibited the expression of ABCG2, MDR1 and MRP1, while increased Bcl-2, Caspase 3 (P < 0.05). However, AKT and p-AKT didn’t show significantly difference between each group (*P* > 0.05)

The effects of JPYW on drug-resistant related proteins ABCG2, AKT, Bcl-2, Caspase3, MDR1, MRP1, P-AKT were detected by immunohistochemical experiments and the results were shown in Fig. 6. Compared with the control group, the expression of ABCG2, MDR1 and MRP1 in JPYW-high group was significantly decreased, while the expression of Bcl-2, Caspase 3 was significantly increased (P < 0.05). Compared with the JPYW-high group, JPYW+ 5-Fu significantly inhibited the expression of ABCG2, MDR1 and MRP1, while increased Bcl-2, Caspase 3 (P < 0.05). However, AKT and p-AKT didn’t show significantly difference between each group (*P* > 0.05).

## Discussion

In this research, the human gastric cancer drug-resistant cell line BGC823/5-Fu was established by repeated low-dose 5-Fu shocks, the drug RI and the proliferation were detected by the MTT assay. Subsequently, the study on the effect of JPYW on the expression of drug-resistant related proteins was carried out and possible mechanisms were explored. It proved that JPYW down-regulated the expression of drug-resistant related gene and proteins, and this effect was closely related to the PI3K/AKT signaling pathway.

Apoptosis induction occupies an important position in the modern cancer chemotherapies, for which the major treatment purpose is to specifically induce apoptosis in malignant tumor cells but have no influence on normal cells [14]. As shown in our research, the apoptosis of JPYW on BGC823 cells was observed by flow cytometry detection and Hoechst fluorescence staining. The BGC823 and BGC823/5-Fu xenograft tumor nude mice models were established and apoptosis was observed by Tunel method. Also, the expressions of caspase-3 were observed by immunohistochemistry. Our results showed that JPYW can induce the apoptosis on BGC823 and BGC823/5-Fu cells both in vitro and in vivo.

Nowadays, drug resistance has been considered as a huge obstacle in cancer treatment, especial for the gastric cancer chemotherapies [15]. In this study, the effects of JPYW on the expression of drug-resistant related proteins MDR1, MRP1 and ABCG2 were carried out. It revealed that JPYW down-regulated the expression of these drug-resistant related proteins. The mRNA expression of MDR1 in BGC823/5-Fu cells was significantly higher than BGC823 cells which indicates the successful establishment of drug-resistant cell line BGC823/5-Fu. The expression of ABCG2, MDR1 and MRP1 in BGC823/5-Fu cells gradually decreased with JPYW concentration accumulated, and JPYW also down-regulated the expression of MDR1, MRP1 and ABCG2 in vivo. This indicated that JPYW could reverse drug resistance both in vitro and in vivo which might have led to a higher apoptosis rate in the JPYW+ 5-Fu group than in the JPYW-high group. Also, our results showed that JPYW down-regulated the ratio of p-AKT expression to AKT expression in vitro, as well as after the combination with pathway inhibitor LY294002. These suggested the inhibitory effect of JPYW on the PI3K/AKT pathway in vitro.

Undeniably, this research is not perfect and still need further studies. Inconsistent with in vitro experiments, in vivo results showed that JPYW didn’t down-regulate AKT or p-AKT expression. In addition, JPYW upregulated the expression of Bcl-2, which is an anti-apoptosis protein involved in PI3K/AKT pathway. And even though the protein expression of AKT or p-Akt was not significantly changed, AKT mRNA expression was decreased in the JPYW-low group and JPYW+ 5-Fu group and increased in the JPYW-high group and 5-Fu group, which indicated further study is needed to figure out the mechanism. These results suggested that although JPYW inhibited the proliferation and reversed drug resistance of BGC823/5-Fu cells both in vitro and in vivo, the mechanisms may be diverse. This phenomenon can possibly be attributed to the changes in the active ingredients of JPYW after in vivo metabolism. And it provided a direction for our researches on the serum composition and in vivo metabolomics of JPYW in the future.

## Conclusions

The effects of JPYW on the expression of drug-resistant related gene and proteins were carried out and possible mechanisms were explored. JPYW can induce the apoptosis of BGC823 and BGC823/5-Fu cells, and down-regulate the expression of MDR1, MRP1 and ABCG2 in vitro and in vivo. The in vitro mechanism is related to the PI3K/AKT signaling pathway.

## Supplementary Information

**Additional file 1.**

## Data Availability

The datasets used and/or analyzed during the current study available from the corresponding author on reasonable request.

## Abbreviations

MTT: Methyl thiazolyl tetrazolium
JPYW: Jianpi Yangwei decoction
MDR1: Multidrug resistance gene 1
MRP1: Multidrug resistance-associated protein 1
ABCG2: ATP binding cassette subfamily G member 2
RI: Resistance index
RT-PCR: Reverse transcription-polymerase chain reaction
TCM: Traditional Chinese Medicine
DMSO: Dimethylsulfoxide
OD: Optical density
IC_50_: Half maximal inhibitory concentration

## References

[1] Xu W, Yang Z, Lu N (2016). Molecular targeted therapy for the treatment of gastric cancer. J Exp Clin Cancer Res.

[2] Holohan C, Van Schaeybroeck S, Longley DB (2013). Cancer drug resistance: an evolving paradigm. Nat Rev Cancer.

[3] Wagner AD, Syn NL, Moehler M (2017). Chemotherapy for advanced gastric cancer. Cochrane Database Syst Rev.

[4] Vasile E, Caparello C, Caponi S (2014). Not only chemotherapy in the second-line treatment of metastatic gastric cancer. Ann Oncol.

[5] Longley DB, Harkin DP, Johnston PG (2003). 5-fluorouracil: mechanisms of action and clinical strategies. Nat Rev Cancer.

[6] Liu P, Wang X, Hu CH (2012). Bioinformatics analysis with graph-based clustering to detect gastric cancer-related pathways. Genet Mol Res.

[7] Kobayashi O, Tsuburaya A, Yoshikawa T (2006). The efficacy of gastrectomy for large gastric cancer. Int J Clin Oncol.

[8] Park SR, Kook MC, Choi IJ (2010). Predictive factors for the efficacy of cetuximab plus chemotherapy as salvage therapy in metastatic gastric cancer patients. Cancer Chemother Pharmacol.

[9] Lv W, Piao JH, Jiang JG (2012). Typical toxic components in traditional Chinese medicine. Expert Opin Drug Saf.

[10] Guo LT, Wang SQ, Su J (2019). Baicalin ameliorates neuroinflammation-induced depressive-like behavior through inhibition of toll-like receptor 4 expression via the PI3K/AKT/FoxO1 pathway. J Neuroinflammation.

[11] Ma YF, Sun X, Nian JY (2018). Thinking and advance in traditional Chinese medicine syndromes research of breast cancer. China J Tradit Chin Med Pharm.

[12] Quan P, Zheng PY, You SF (2016). Clinical and psychometric validation of the quality of life assessment system for advanced gastric cancer based on traditional Chinese medicine. Chin J Integr Med.

[13] Shu P, Tang H, Zhou B (2019). Effect of Yiqi Huayu Jiedu decoction on stages II and III gastric cancer: a multicenter, prospective, cohort study. Medicine..

[14] Xie ZH, Quan MF, Liu F (2011). 5-allyl-7-gen-difluoromethoxychrysin enhances TRAIL-induced apoptosis in human lung carcinoma A549 cells. BMC Cancer.

[15] Wang JP, Li JH, Wang KS (2009). Advances in mechanisms of drug resistance in gastric cancer. World Chinese J Digestol.

