# Supplementary figures and images for: Jianpi Yangwei decoction promotes apoptosis and suppresses proliferation of 5-fluorouracil resistant gastric cancer cells in vitro and in vivo
Source: BMC Complement Med Ther. 2020 Nov 10;20:337. doi: 10.1186/s12906-020-03135-8 (PMC7654161; doi:10.1186/s12906-020-03135-8)

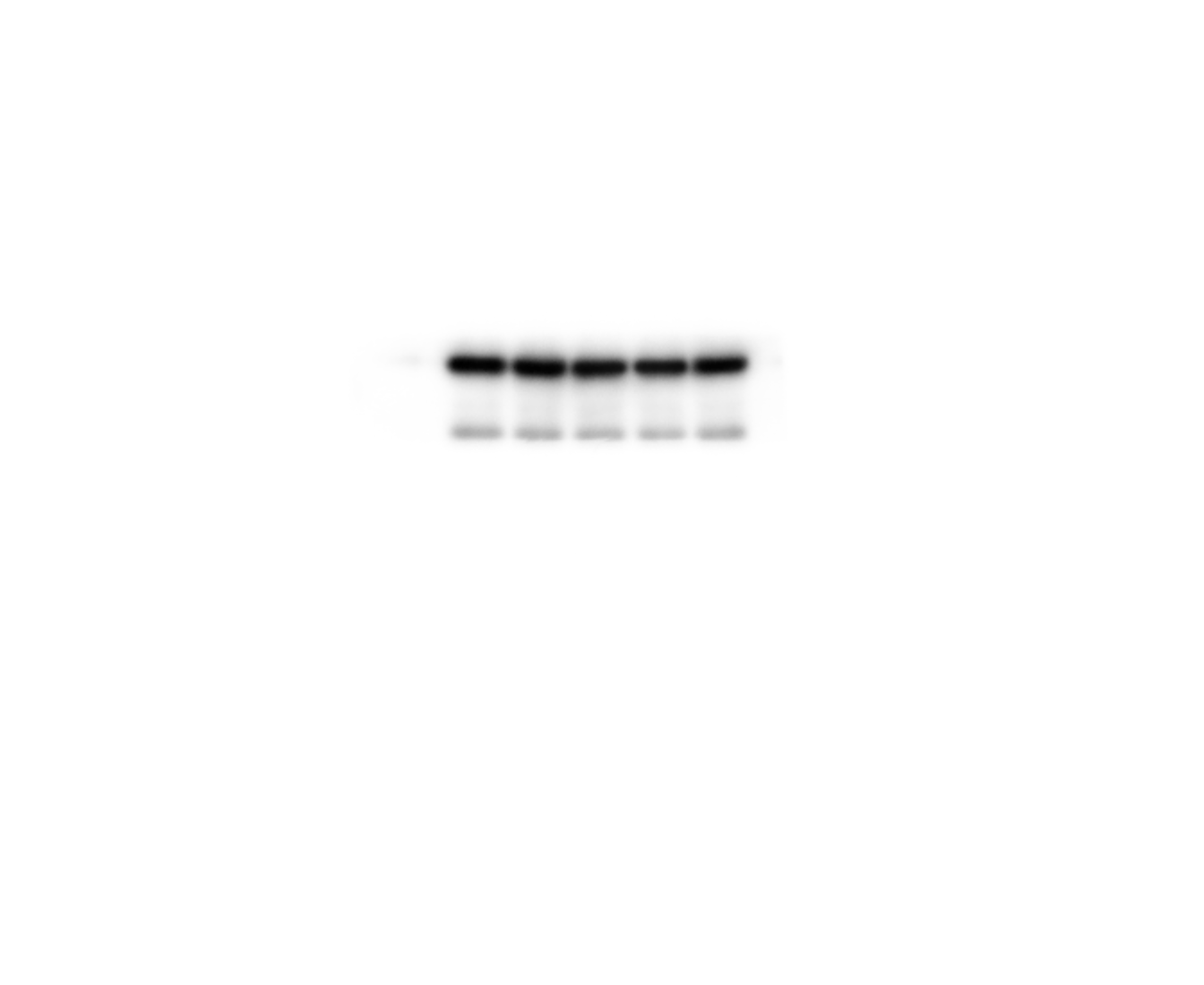

Supplement: Supplementary file 1 — Additional file 1. [file 12906_2020_3135_MOESM1_ESM.zip › JPYW Actin 2R3.tif]

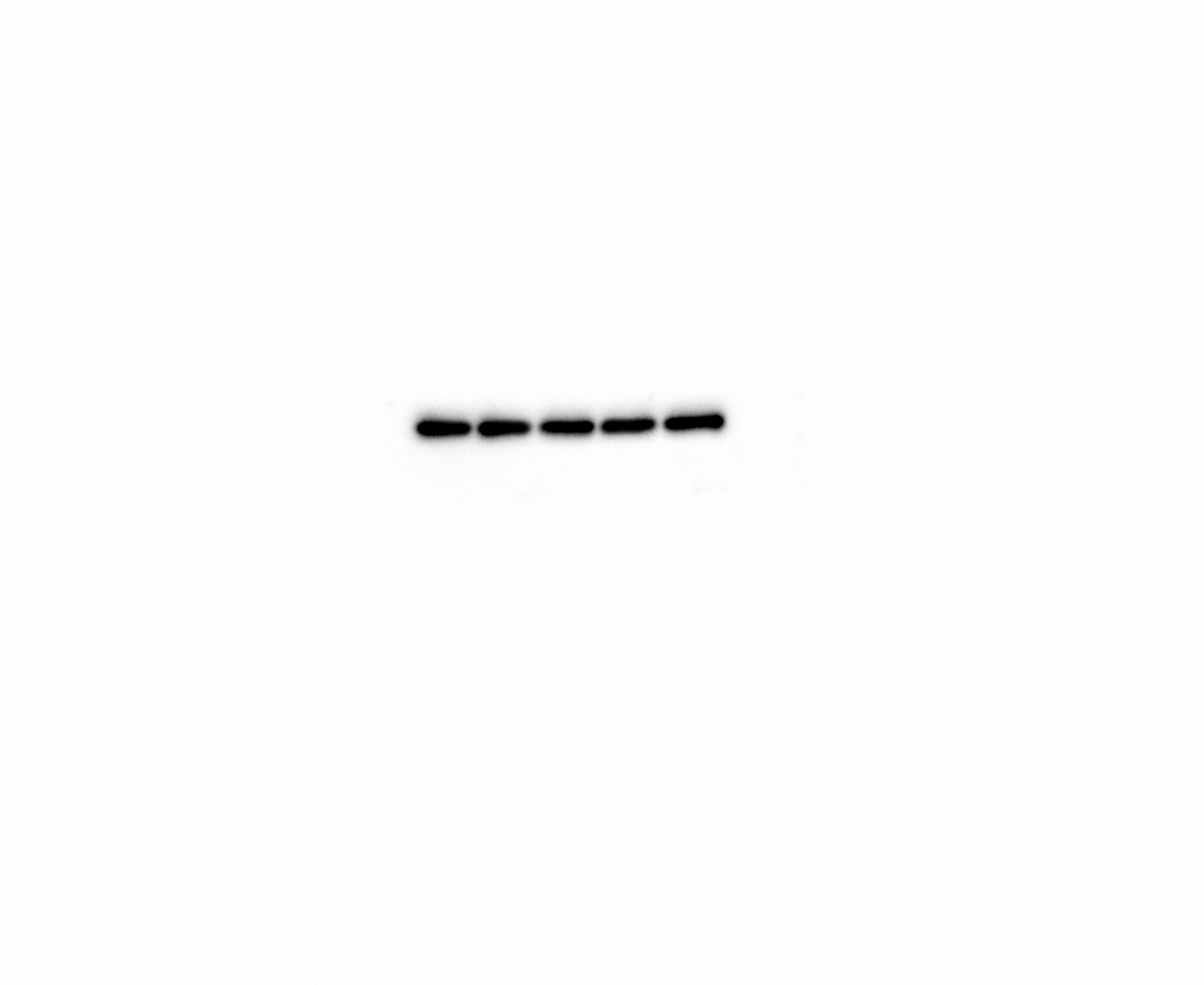

Supplement: Supplementary file 1 — Additional file 1. [file 12906_2020_3135_MOESM1_ESM.zip › JPYW actin 3R3.tif]

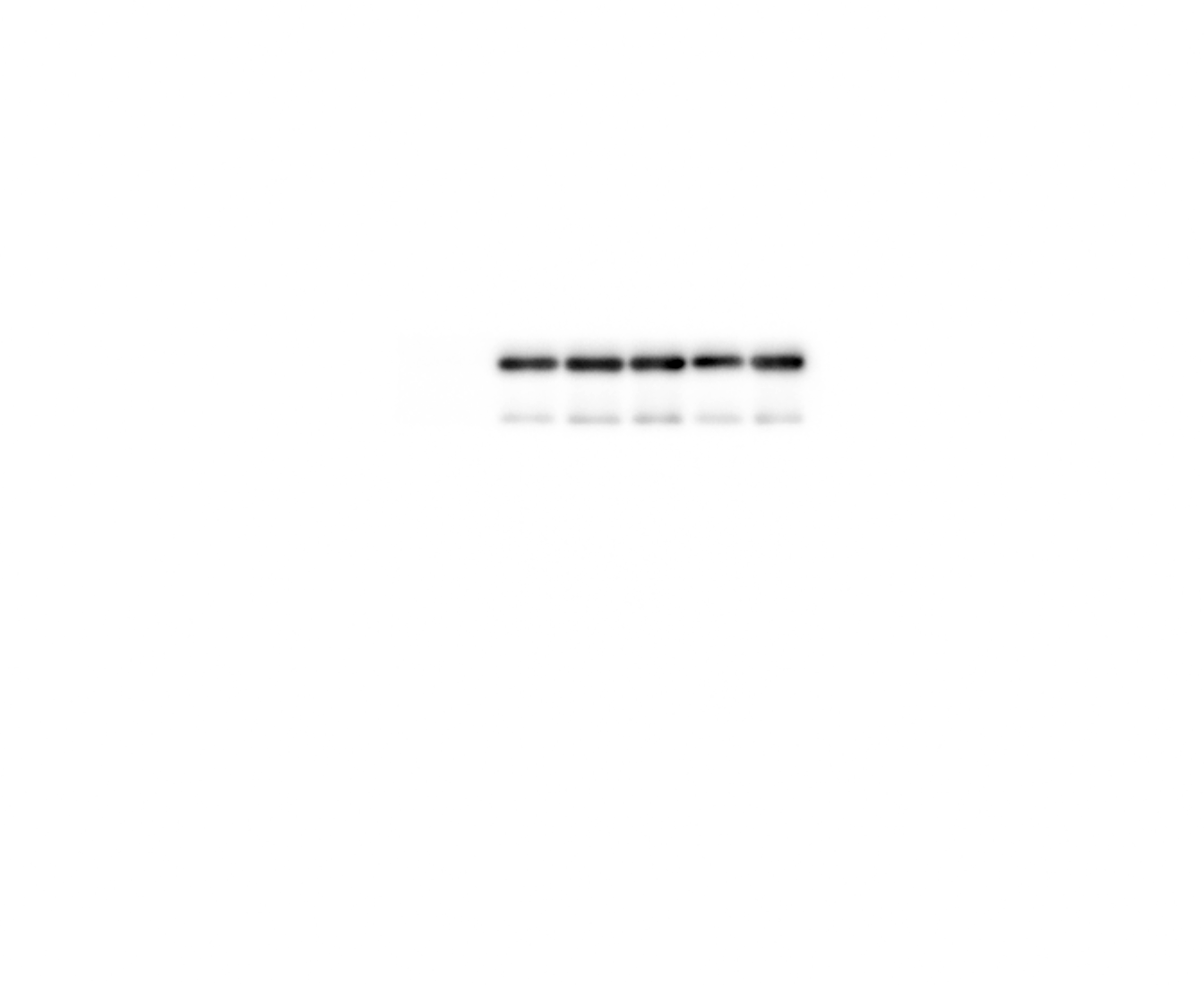

Supplement: Supplementary file 1 — Additional file 1. [file 12906_2020_3135_MOESM1_ESM.zip › JPYW AKT 1R3.tif]

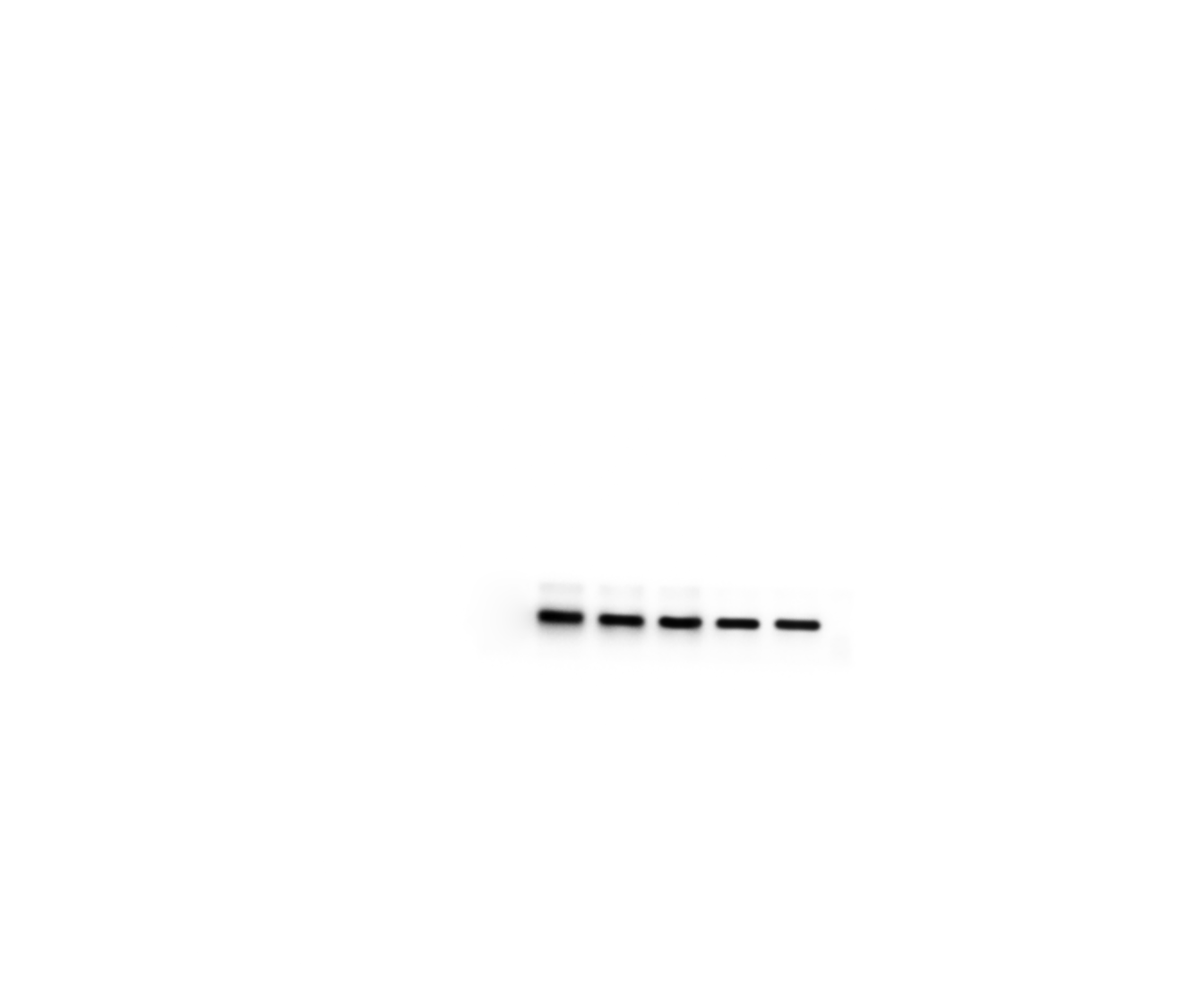

Supplement: Supplementary file 1 — Additional file 1. [file 12906_2020_3135_MOESM1_ESM.zip › JPYW AKT 2R3.tif]

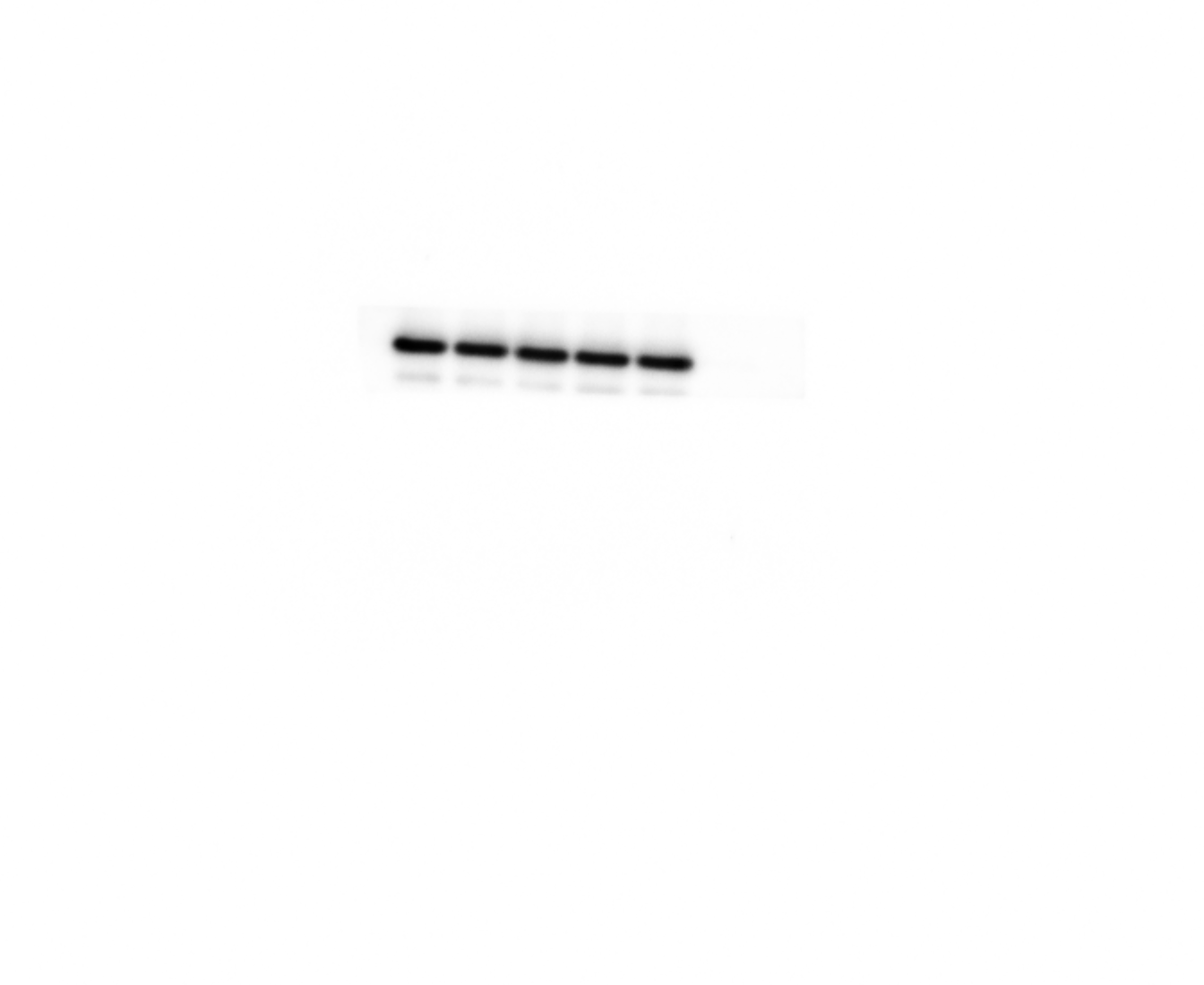

Supplement: Supplementary file 1 — Additional file 1. [file 12906_2020_3135_MOESM1_ESM.zip › JPYW AKT 3R3.tif]

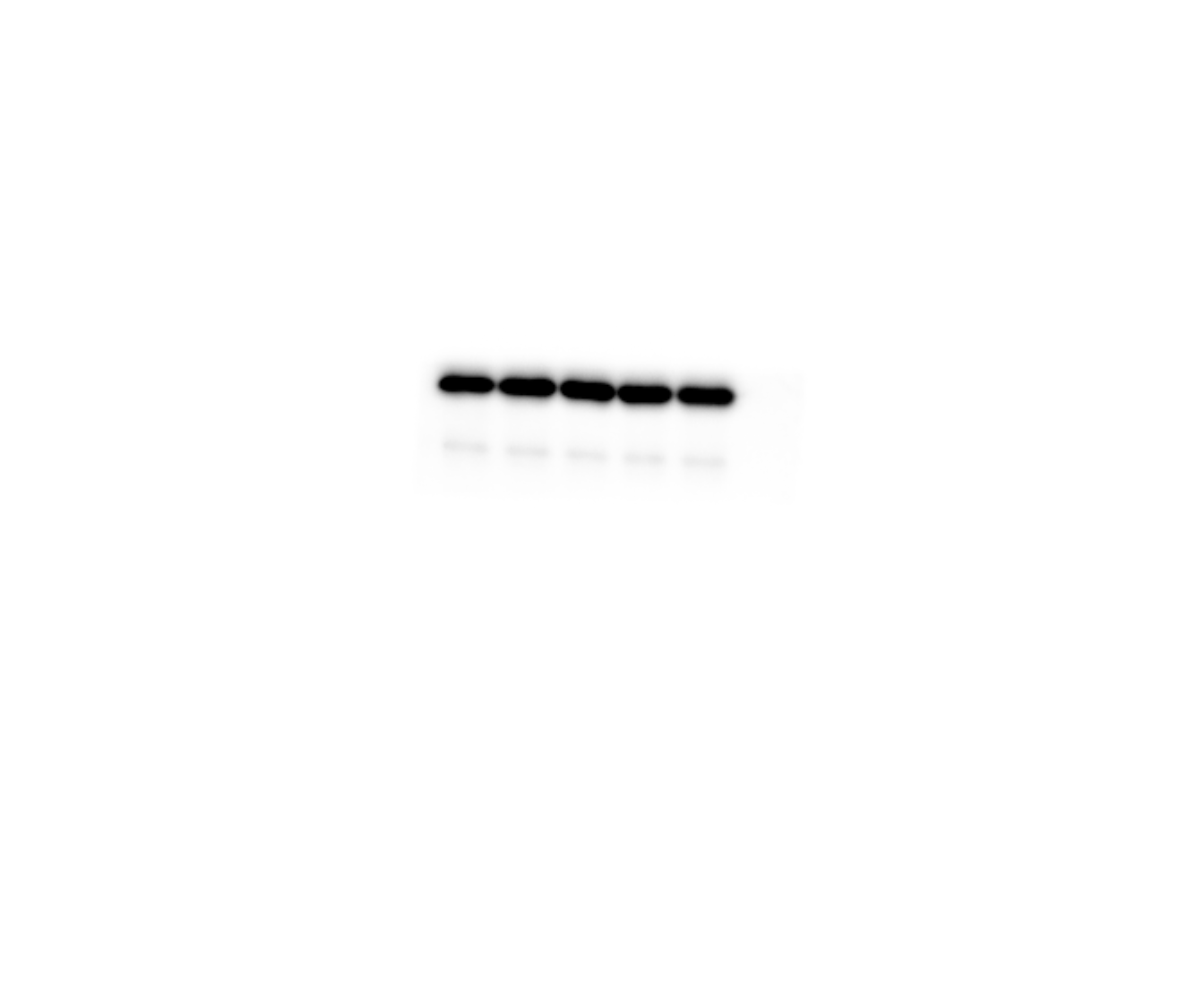

Supplement: Supplementary file 1 — Additional file 1. [file 12906_2020_3135_MOESM1_ESM.zip › JPYW GAPDH 1R3.tif]

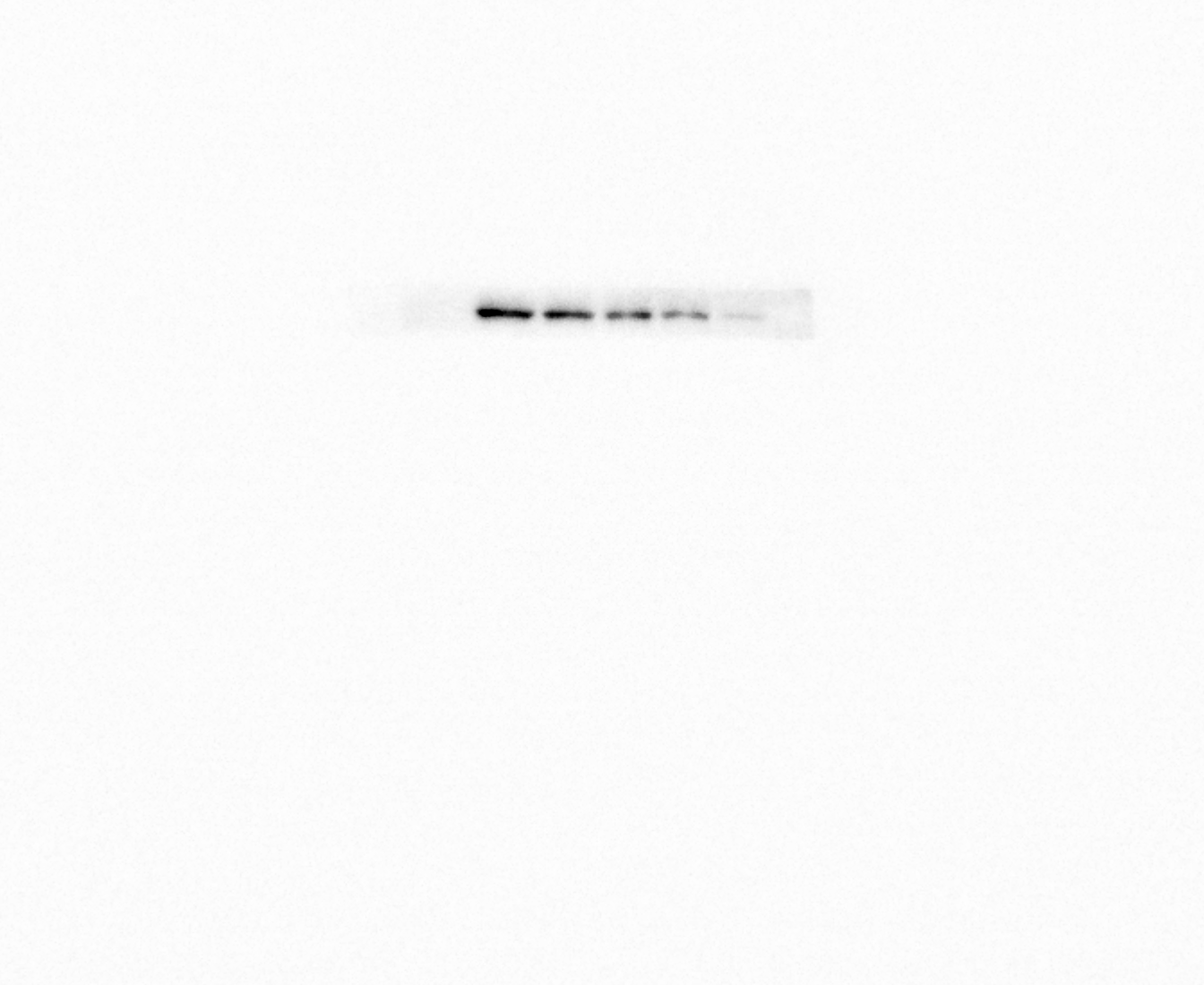

Supplement: Supplementary file 1 — Additional file 1. [file 12906_2020_3135_MOESM1_ESM.zip › JPYW P-AKT 1R3.tif]

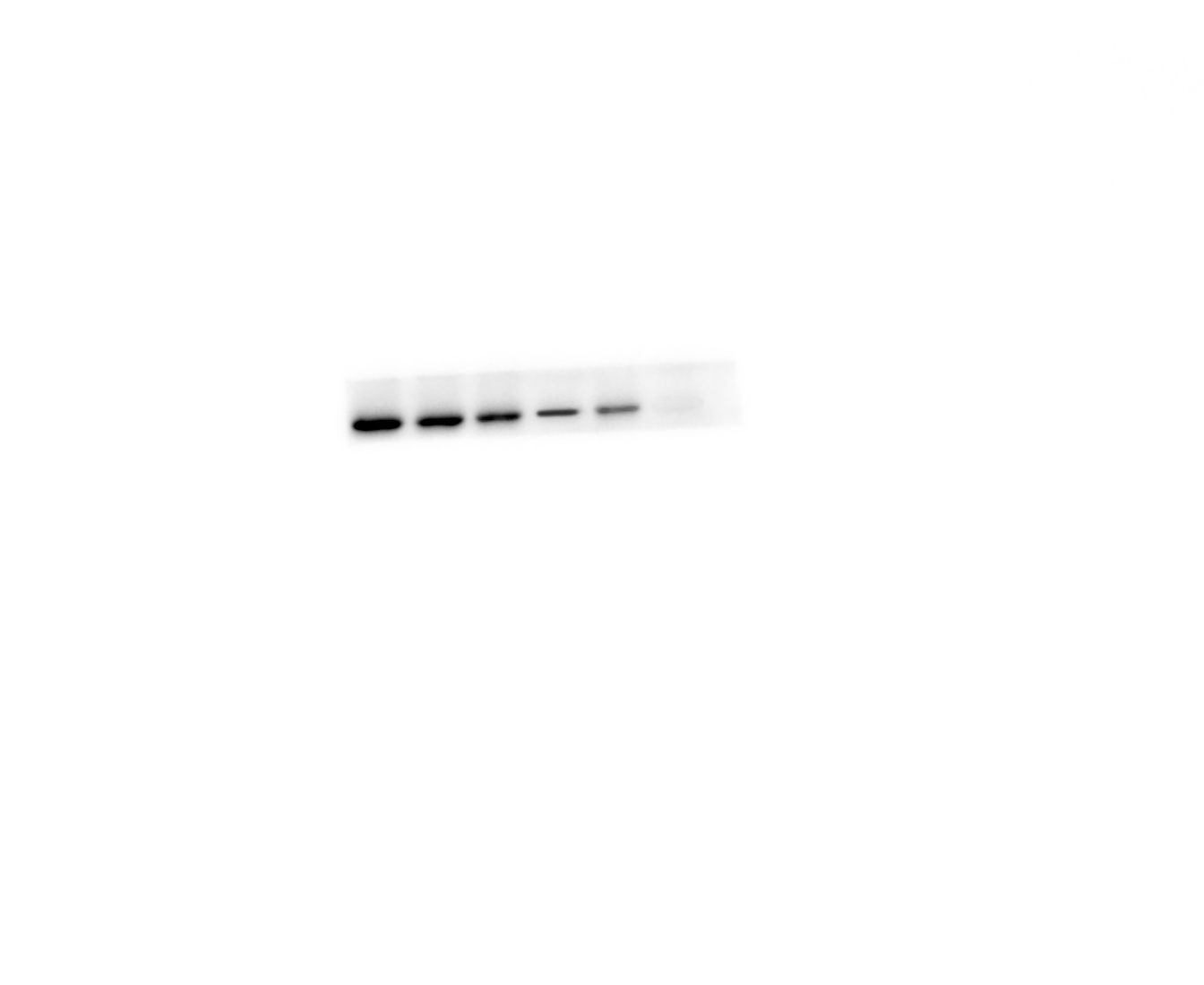

Supplement: Supplementary file 1 — Additional file 1. [file 12906_2020_3135_MOESM1_ESM.zip › JPYW p-AKT 2R3.tif]

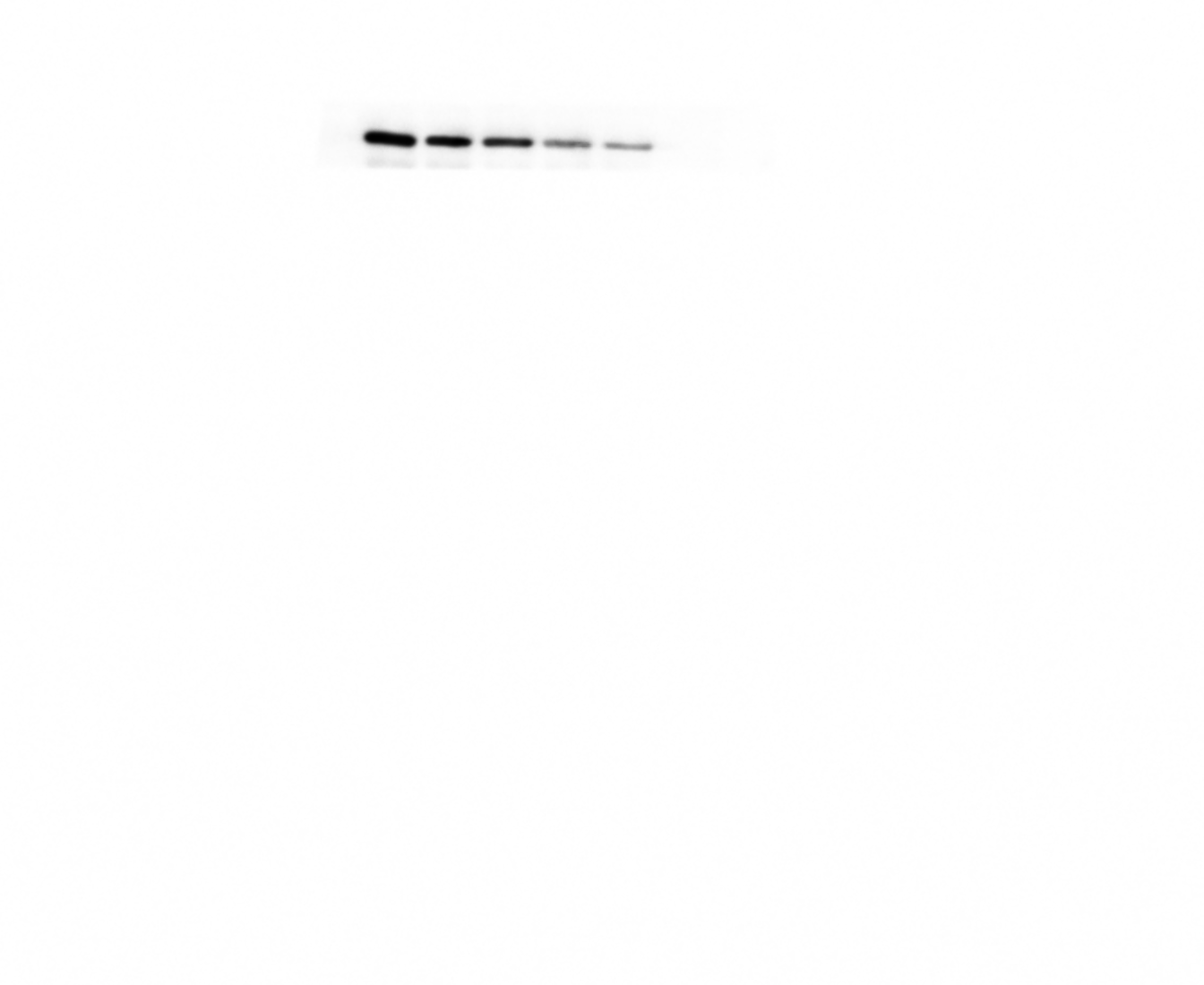

Supplement: Supplementary file 1 — Additional file 1. [file 12906_2020_3135_MOESM1_ESM.zip › JPYW p-AKT 3R3.tif]

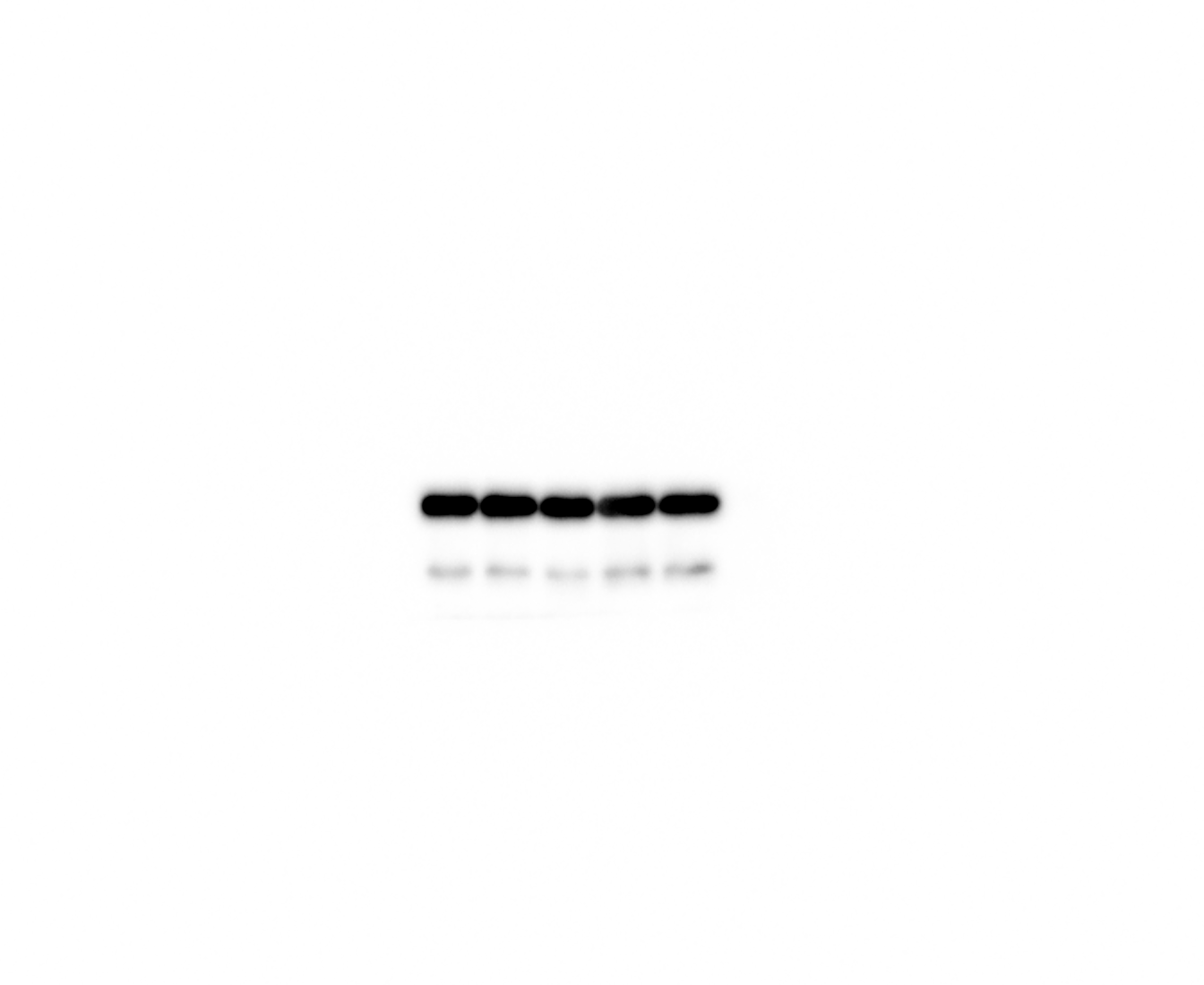

Supplement: Supplementary file 1 — Additional file 1. [file 12906_2020_3135_MOESM1_ESM.zip › JPYW+LY294002 actin 2R3.tif]

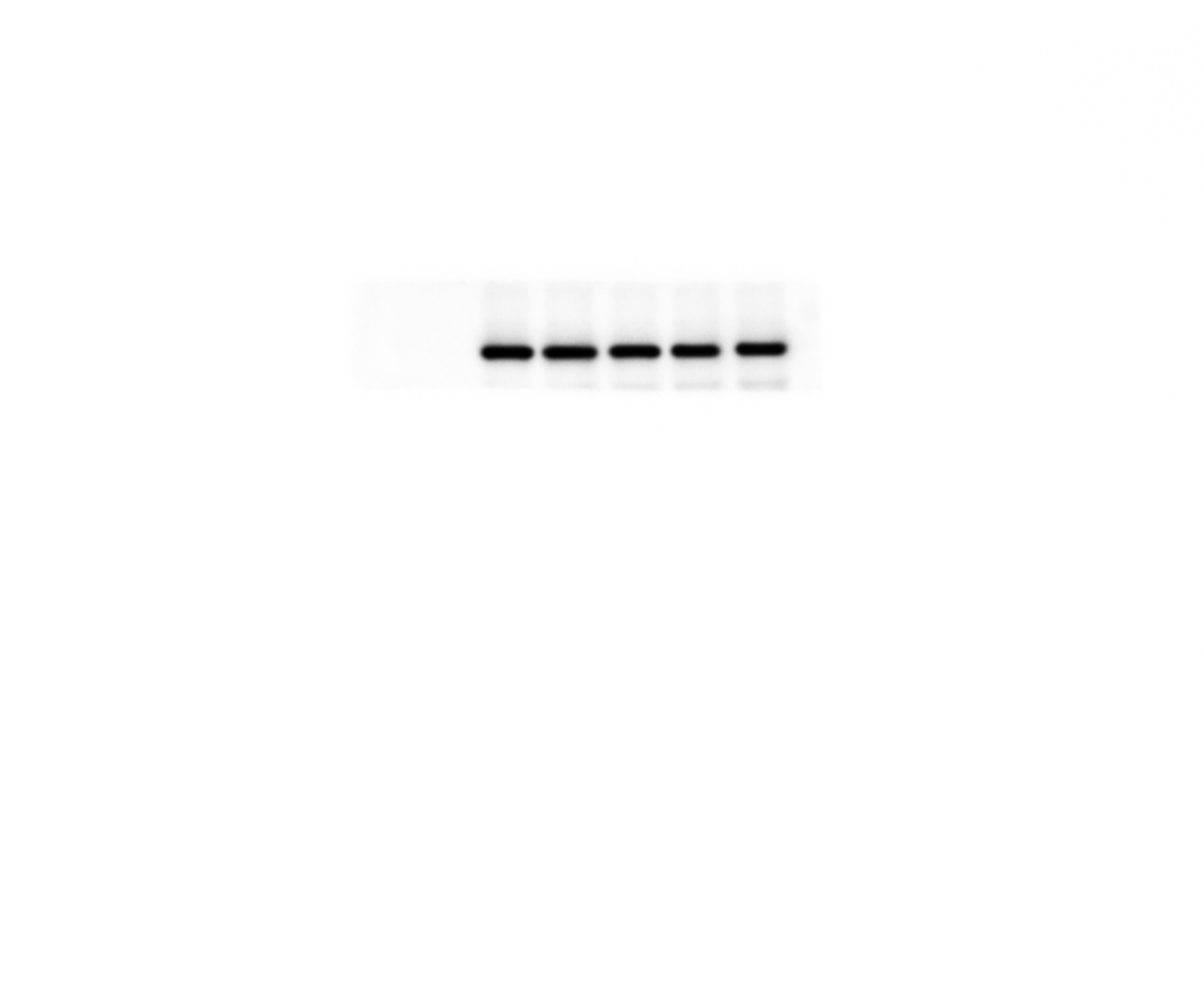

Supplement: Supplementary file 1 — Additional file 1. [file 12906_2020_3135_MOESM1_ESM.zip › JPYW+LY294002 Actin 3R3.tif]

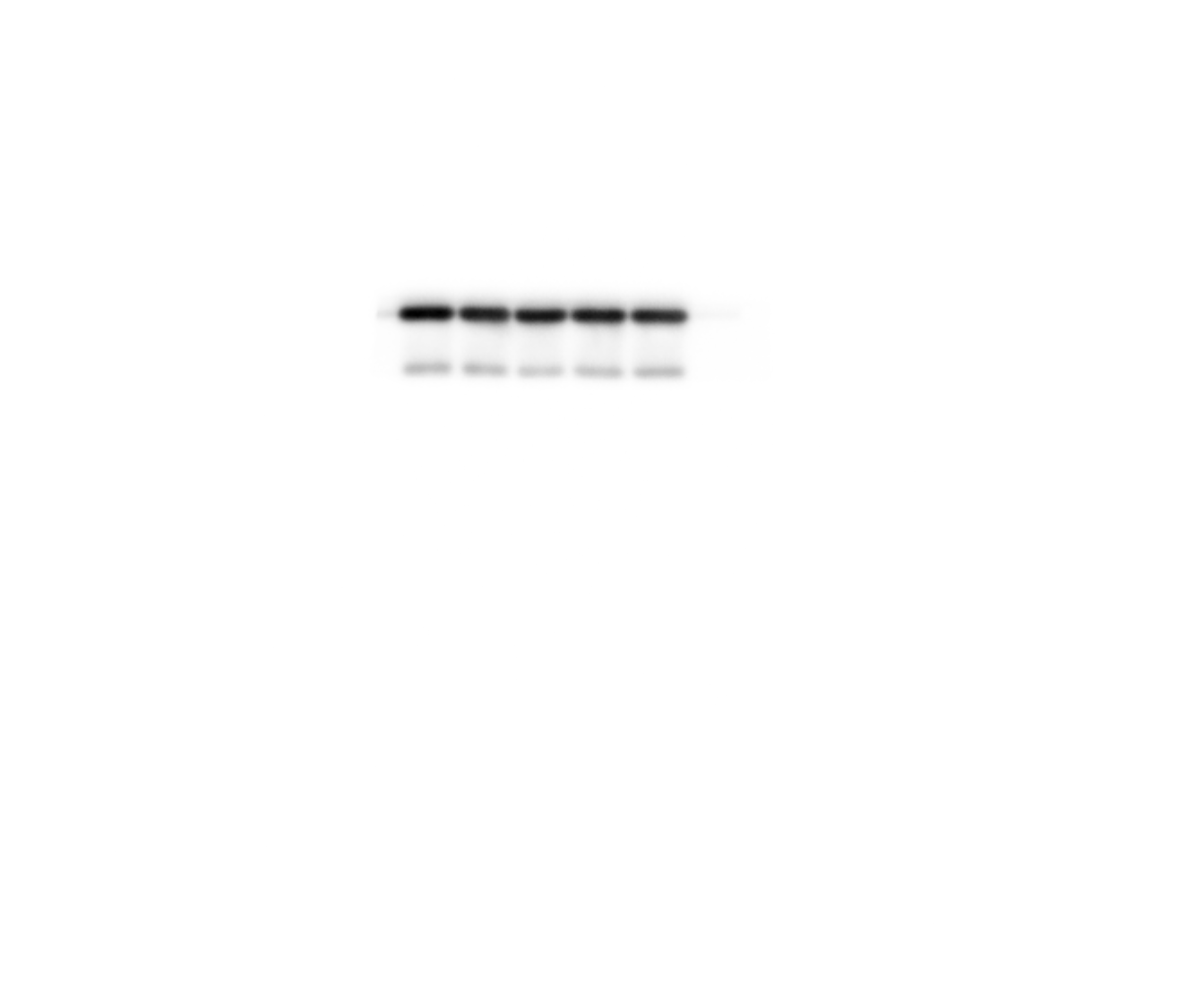

Supplement: Supplementary file 1 — Additional file 1. [file 12906_2020_3135_MOESM1_ESM.zip › JPYW+LY294002 AKT 1R3.tif]

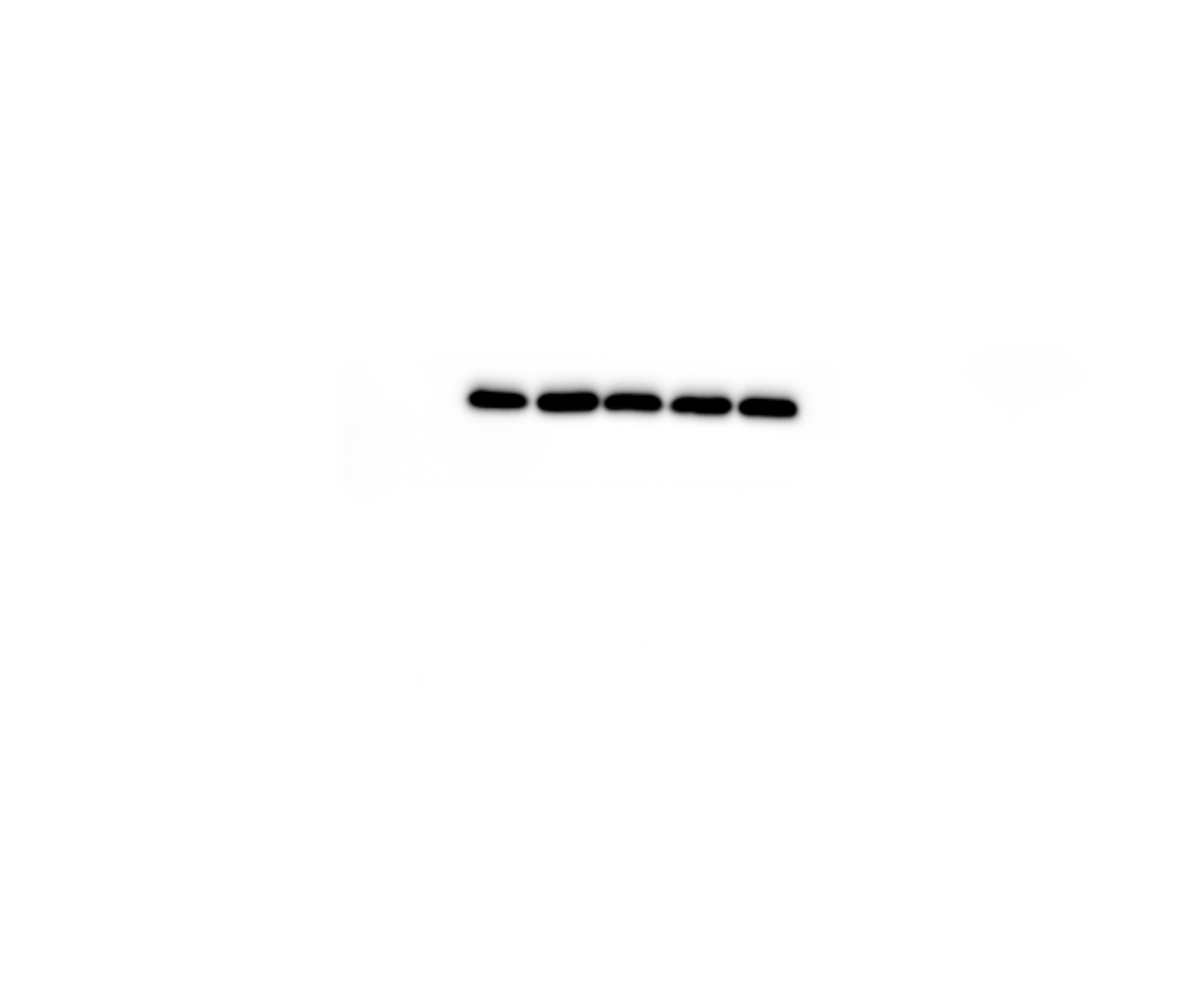

Supplement: Supplementary file 1 — Additional file 1. [file 12906_2020_3135_MOESM1_ESM.zip › JPYW+LY294002 AKT 2R3.tif]

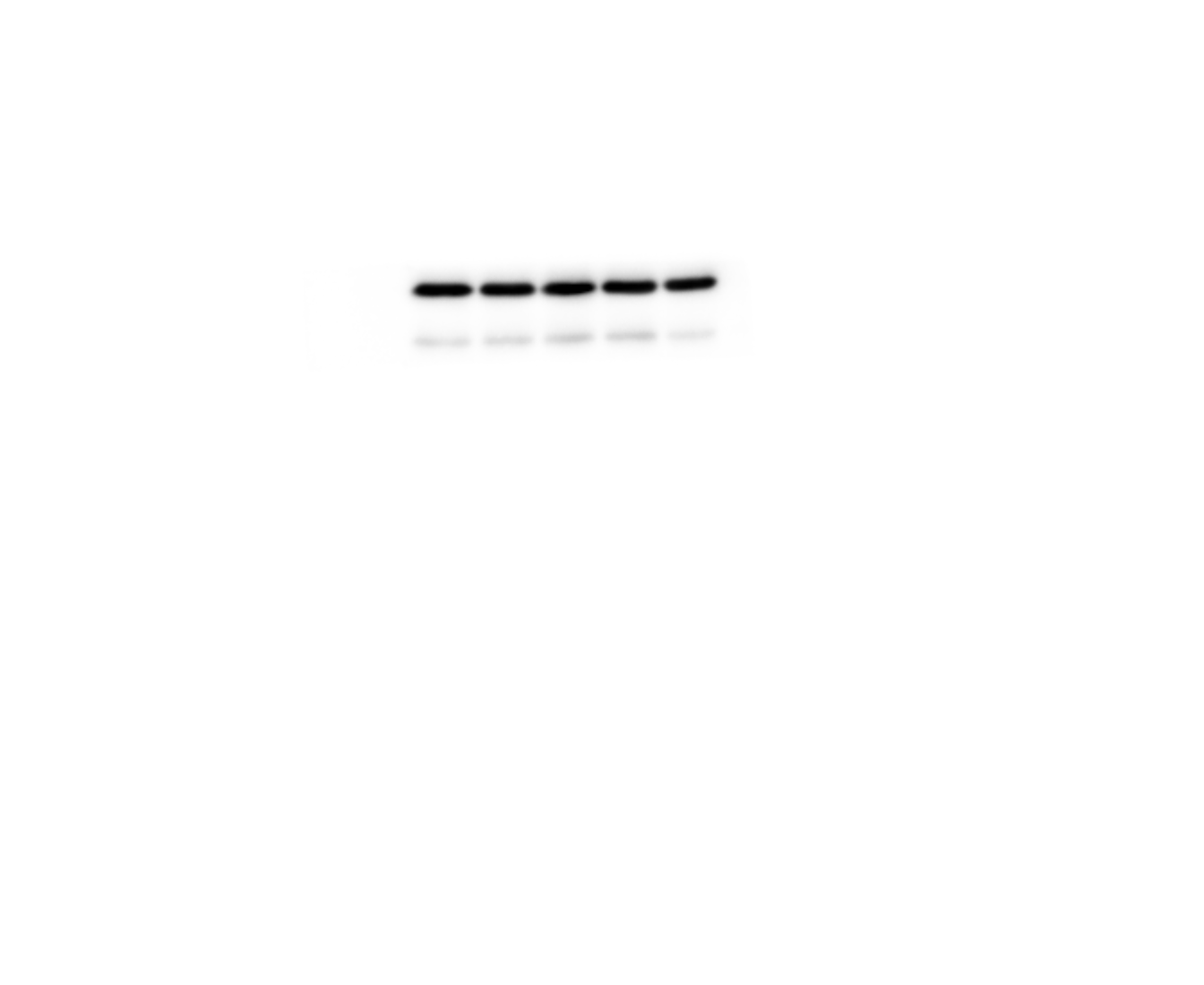

Supplement: Supplementary file 1 — Additional file 1. [file 12906_2020_3135_MOESM1_ESM.zip › JPYW+LY294002 AKT 3R3.tif]

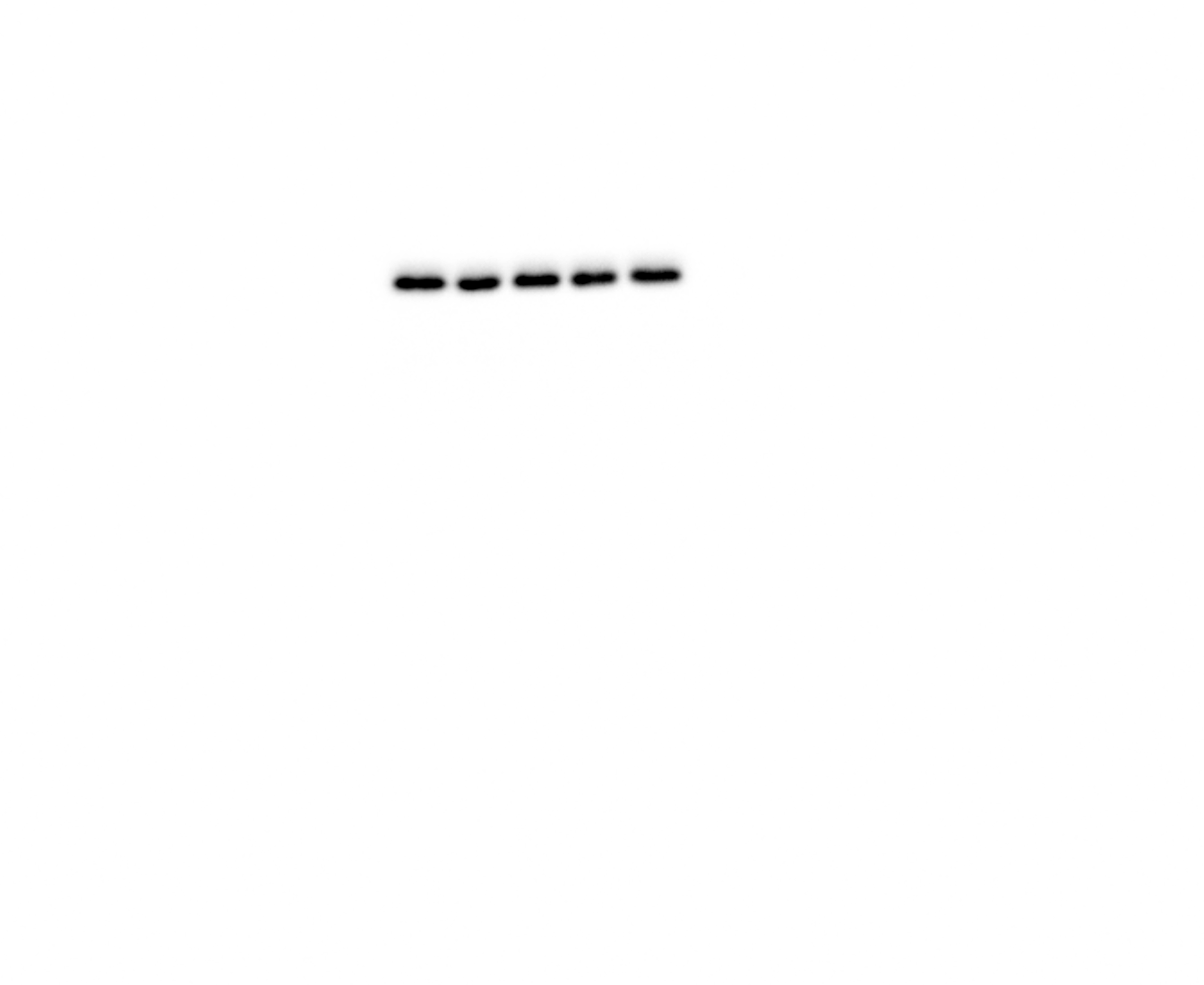

Supplement: Supplementary file 1 — Additional file 1. [file 12906_2020_3135_MOESM1_ESM.zip › JPYW+LY294002 GAPDH 1R3.tif]

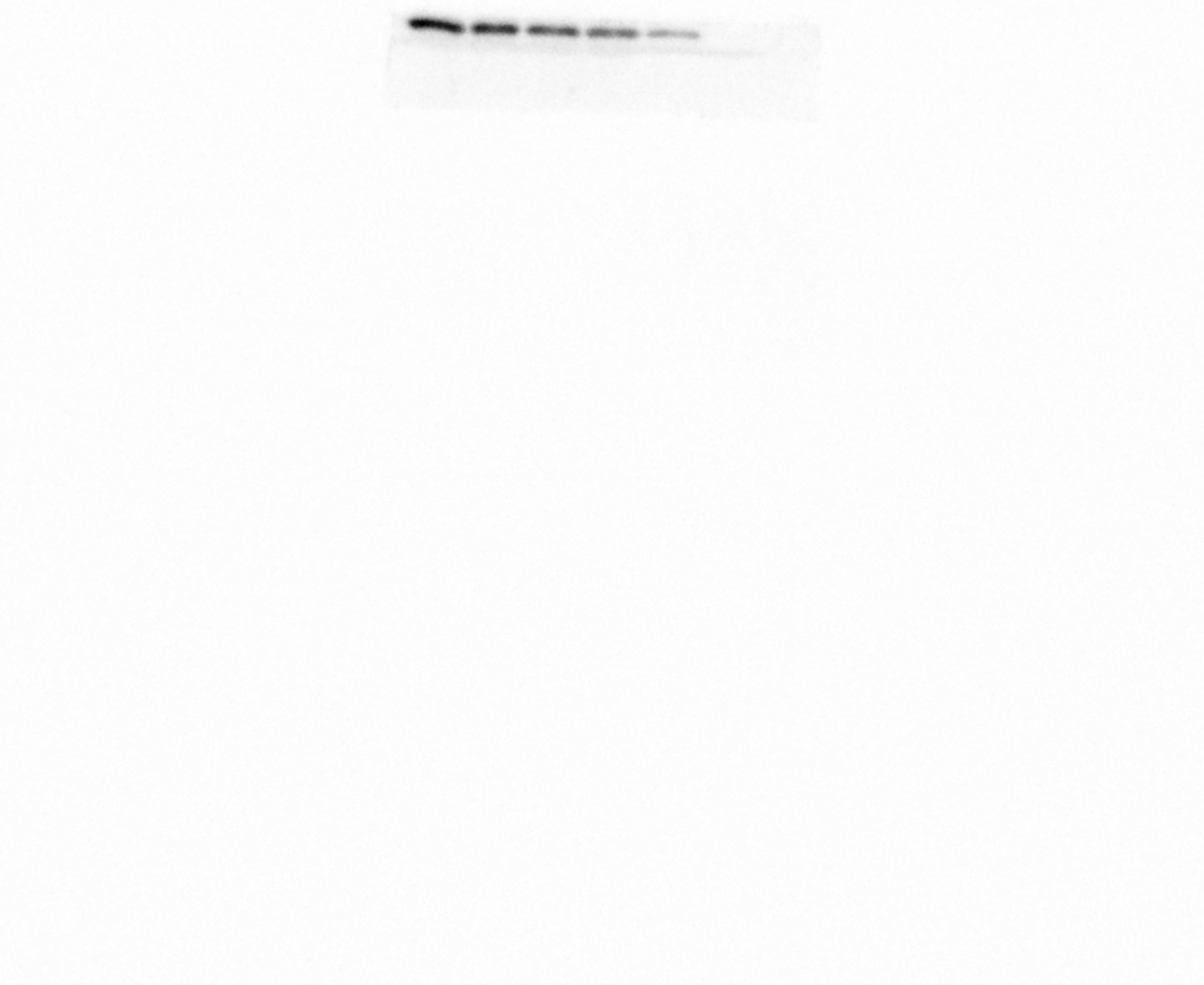

Supplement: Supplementary file 1 — Additional file 1. [file 12906_2020_3135_MOESM1_ESM.zip › JPYW+LY294002 P-AKT 1R3.tif]

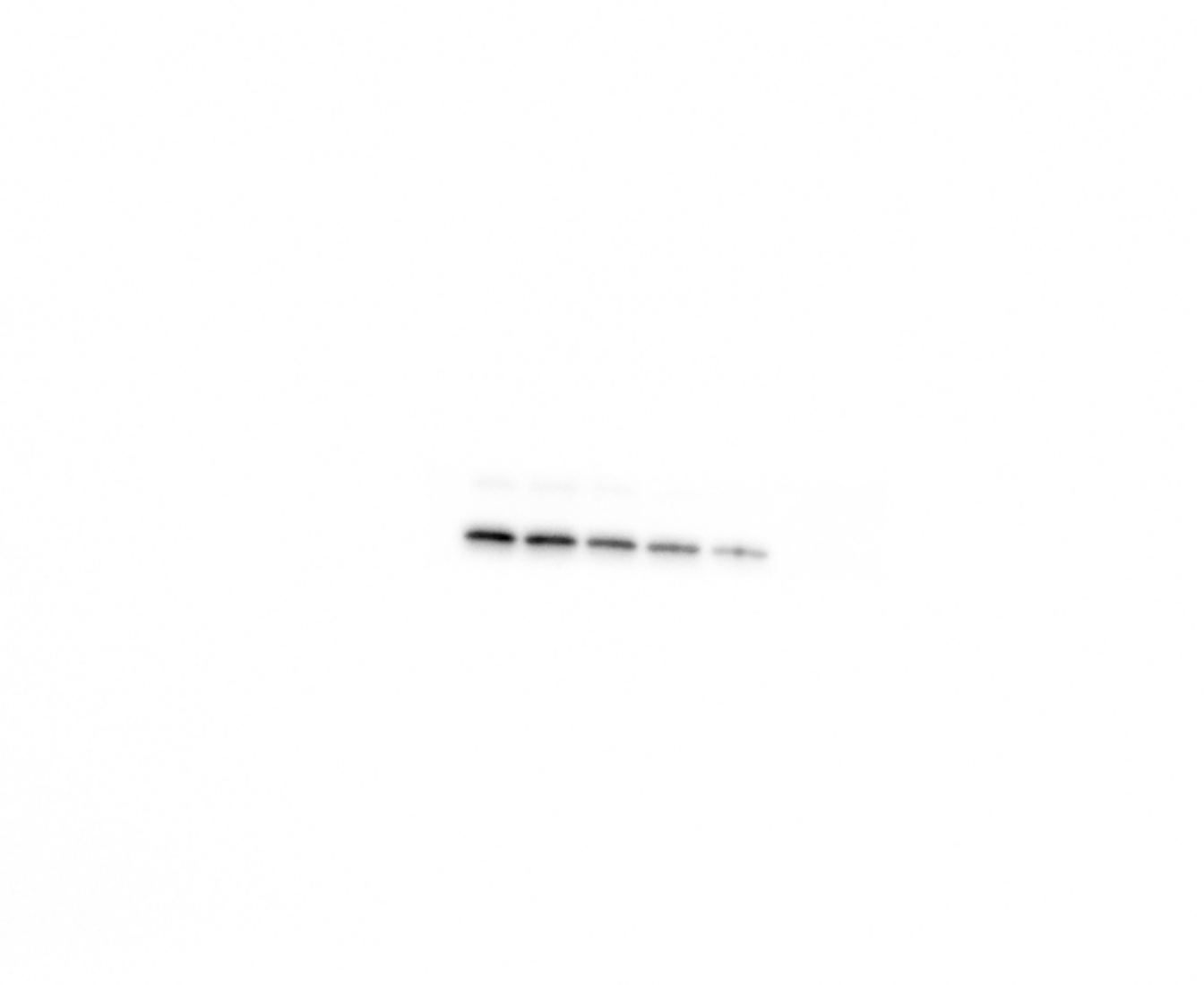

Supplement: Supplementary file 1 — Additional file 1. [file 12906_2020_3135_MOESM1_ESM.zip › JPYW+LY294002 p-AKT 2R3.tif]

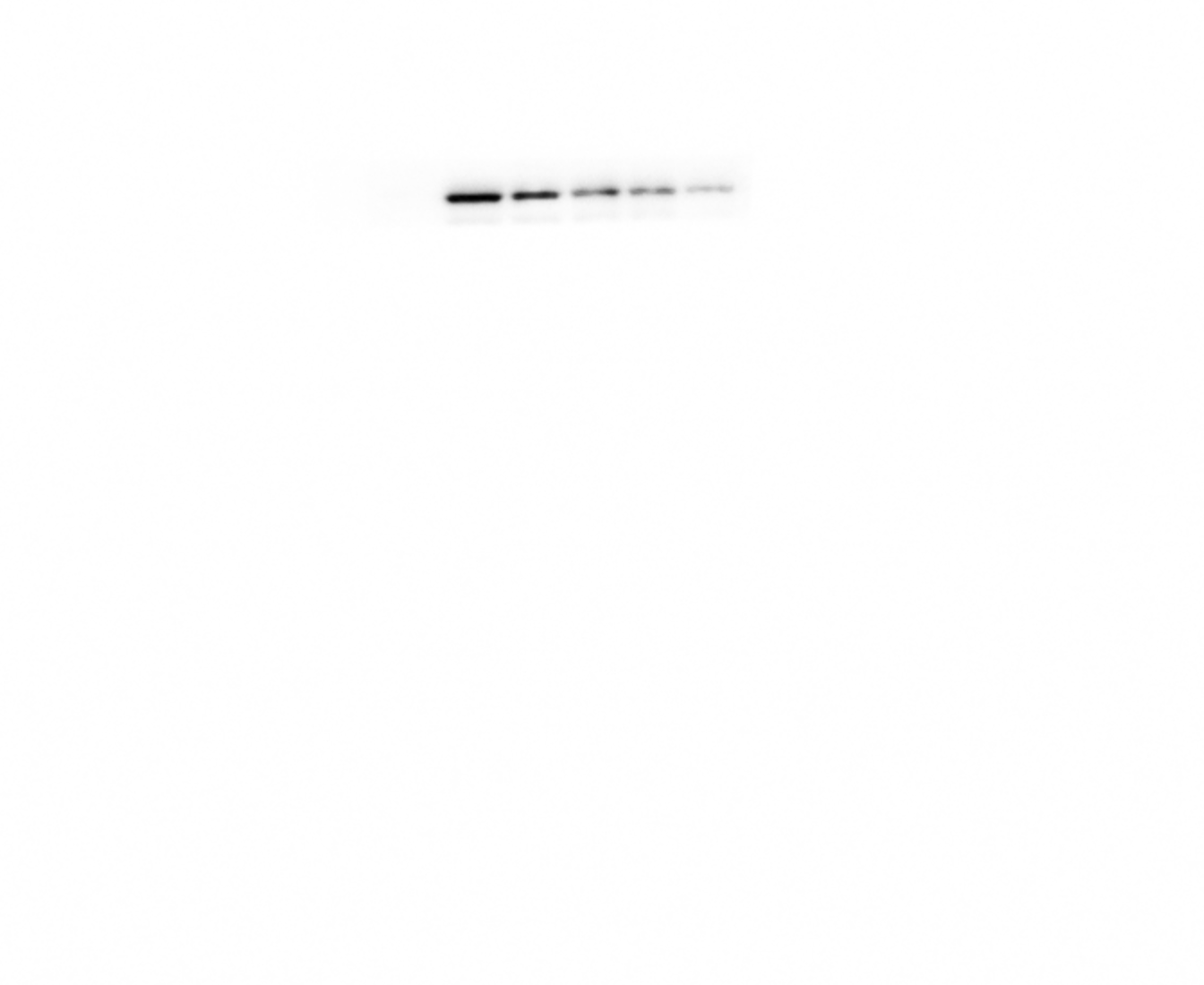

Supplement: Supplementary file 1 — Additional file 1. [file 12906_2020_3135_MOESM1_ESM.zip › JPYW+LY294002 p-AKT 3R3.tif]
